# Protective Properties of Radio-Chemoresistant Glioblastoma Stem Cell Clones Are Associated with Metabolic Adaptation to Reduced Glucose Dependence

**DOI:** 10.1371/journal.pone.0080397

**Published:** 2013-11-18

**Authors:** Fei Ye, Yibei Zhang, Yue Liu, Kazunari Yamada, Jonathan L. Tso, Jimmy C. Menjivar, Jane Y. Tian, William H. Yong, Dörthe Schaue, Paul S. Mischel, Timothy F. Cloughesy, Stanley F. Nelson, Linda M. Liau, William McBride, Cho-Lea Tso

**Affiliations:** 1 Department of Surgery/Surgical Oncology, David Geffen School of Medicine, University of California Los Angeles, Los Angeles, California, United States of America; 2 Department of Neurosurgery, Tongji Hospital, Tongji Medical College, Huazhong University of Science and Technology, Wuhan, Hubei, China; 3 Department of Orthopedics, Zhongshan Hospital, Xiamen University, Xiamen, Fujian, China; 4 Department of Advanced Molecular and Cell Therapy, Kyushu University Hospital, Higashi-ku, Fukuoka, Japan; 5 Department of Pathology and Laboratory Medicine, David Geffen School of Medicine, University of California Los Angeles, Los Angeles, California, United States of America; 6 Department of Radiation-Oncology, David Geffen School of Medicine, University of California Los Angeles, Los Angeles, California, United States of America; 7 Department of Neurology, David Geffen School of Medicine, University of California Los Angeles, Los Angeles, California, United States of America; 8 Department of Human Genetics, David Geffen School of Medicine, University of California Los Angeles, Los Angeles, California, United States of America; 9 Department of Neurosurgery, David Geffen School of Medicine, University of California Los Angeles, Los Angeles, California, United States of America; 10 Jonsson Comprehensive Cancer Center, University of California Los Angeles, Los Angeles, California, United States of America; Cleveland Clinic, United States of America

## Abstract

Glioblastoma stem cells (GSC) are a significant cell model for explaining brain tumor recurrence. However, mechanisms underlying their radiochemoresistance remain obscure. Here we show that most clonogenic cells in GSC cultures are sensitive to radiation treatment (RT) with or without temozolomide (TMZ). Only a few single cells survive treatment and regain their self-repopulating capacity. Cells re-populated from treatment-resistant GSC clones contain more clonogenic cells compared to those grown from treatment-sensitive GSC clones, and repeated treatment cycles rapidly enriched clonogenic survival. When compared to sensitive clones, resistant clones exhibited slower tumor development in animals. Upregulated genes identified in resistant clones via comparative expression microarray analysis characterized cells under metabolic stress, including blocked glucose uptake, impaired insulin/Akt signaling, enhanced lipid catabolism and oxidative stress, and suppressed growth and inflammation. Moreover, many upregulated genes highlighted maintenance and repair activities, including detoxifying lipid peroxidation products, activating lysosomal autophagy/ubiquitin-proteasome pathways, and enhancing telomere maintenance and DNA repair, closely resembling the anti-aging effects of caloric/glucose restriction (CR/GR), a nutritional intervention that is known to increase lifespan and stress resistance in model organisms. Although treatment–introduced genetic mutations were detected in resistant clones, all resistant and sensitive clones were subclassified to either proneural (PN) or mesenchymal (MES) glioblastoma subtype based on their expression profiles. Functional assays demonstrated the association of treatment resistance with energy stress, including reduced glucose uptake, fatty acid oxidation (FAO)-dependent ATP maintenance, elevated reactive oxygen species (ROS) production and autophagic activity, and increased AMPK activity and NAD^+^ levels accompanied by upregulated mRNA levels of SIRT1/PGC-1α axis and DNA repair genes. These data support the view that treatment resistance may arise from quiescent GSC exhibiting a GR-like phenotype, and suggest that targeting stress response pathways of resistant GSC may provide a novel strategy in combination with standard treatment for glioblastoma.

## Introduction

Glioblastoma (World Health Organization/WHO grade IV) is the most common and aggressive type of primary malignant brain tumor in adults, killing nearly every patient within two years. Currently, the best standard treatment for newly diagnosed glioblastoma is maximal safe surgical resection followed by radiation treatment (RT) combined with concomitant and adjuvant temozolomide (TMZ) [1]. Although patients whose tumors have a methylated promoter for the gene encoding for O-6-methylguanine-DNA methyltransferase (MGMT) are more likely to benefit from the addition of TMZ to RT, they become resistant to the treatment. The development of resistance suggests that there is a remnant of cancer cells possessing tumorigenic capacity with extraordinary defense mechanisms, enabling them to survive treatment.

Glioblastoma stem cells (GSC) have become a significant experimental model for explaining tumor recurrence because they possess a tumorigenic capacity [2]–[7], a highly migratory nature [7], [8], and a radiochemoresistant phenotype [9]–[11]. The definition of GSC varies with the laboratory, but it is generally accepted that they are a small subset of glioblastoma cells residing within the tumor mass that expresses normal stem cell markers, are capable of clonally growing as tumor spheres *in vitro*, and are able to reconstitute a tumor in mouse brain that recapitulates the histopathological features of the patient tumor from which the GSC were derived. Multiple intrinsic mechanisms underlying resistance to standard treatment in GSC have been proposed, including preferential activation of DNA damage checkpoint response and DNA repair pathway [9], [12], expression of the constitutively active Notch/PI3K/Akt, Wnt, and IGFBP2 signaling pathways [13]–[15], and high expression of anti-apoptotic proteins and drug efflux transporters [16]–[18]. However, some authors did not find different DNA repair mechanisms in stem and non-stem glioma cells [19], [20].

The development of radioresistance or chemoresistance may be considered a cell survival adaptive response (AR) or a hormetic response (HR), where cells become more resistant to stress damage by prior exposure to a low dose of ionizing radiation (IR) or other DNA-damaging agents [21], [22]. AR can also be induced by ROS, which are generated in cells during cellular respiratory metabolism and/or after exposure to IR, and produce low levels of macromolecular damage which includes oxidative stress [23]. Several defense mechanisms underlying radioadaptive protection have been postulated, including enhancement of free radical detoxification, activation of DNA repair systems, induction of new proteins for repair and maintenance, and increase in anti-oxidant production [23]–[25]. Similar to AR/HR in principle, protective effects can be induced by caloric/dietary restriction (CR/DR), a potent nutritional intervention that has been shown to extend the lifespan of multiple species and model organisms for slowing the aging process down, and protect against age-related diseases in humans [26], [27]. Many different mechanisms have been proposed to promote the anti-aging/anti-senescence effects of CR, including disruption of the insulin/insulin-like growth factor-1 (IGF-1) signaling (IIS) pathway [28], attenuation of TOR signaling [29], growth retardation [30], suppression of inflammation [31], mitochondrial hormesis [32], enhancement of autophagy [33], activation of SIRT1 (silent mating type information regulation 2, homolog 1)- PGC-1α (Peroxisome proliferator-activated receptor gamma coactivator-1 alpha) signaling axis [34], [35], enhancement of DNA repair [36], [37], and the hormetic effects of mild stress [38], [39]. Although the effectors which directly contribute to the survival and longevity-enhancing effects of CR are not completely understood, the collective actions of these pathways seem to point towards the generation of metabolic adaptations to nutritional stress, leading to slowed cell growth and activated repair and maintenance networks.

In this study, we explored the potential mechanisms underlying treatment resistance of glioblastoma. We isolated and characterized tumorigenic GSC clones that survived radiochemotherapy. We found that under no glucose deprivation condition, these resistant GSC clones favor the FAO pathway and express a GR-like phenotype, and exhibit reduced glucose uptake, promoted lipid metabolism in mitochondria, increased formation of ROS, and enhanced autophagy, AMP-activated protein kinase (AMPK)-SIRT1 signaling, and upregulated genes associated with global DNA repair activity. These findings could impact the design of more effective therapies aiming to prevent tumor recurrence.

## Materials and Methods

### Glioblastoma sphere cultures

Glioblastoma tumor specimens were obtained from patients who underwent surgery at Ronald Reagan UCLA Medical Center. All samples collected were under patients' written consent, and were approved by the UCLA Institutional Review Board (IRB # 0304-053). GSC culture lines were established from fresh tumors. GSC culture lines were established from fresh tumors. Briefly, tumors were enzyme-digested and washed, followed by red blood cell lysis of the pellet. Dissociated tumor cells were plated and maintained in serum-free DMEM/Ham's F-12 supplemented with 20 ng/ml epidermal growth factor (EGF, Sigma), 20 ng/ml basic fibroblast growth factor (bFGF, Millipore), 10 ng/ml leukemia inhibitory factor (LIF, Millipore), and 1x B27 without vitamin A (Invitrogen). The tumor spheres were dissociated and replated at clonal density and continually passaged until the clonogenic cells were stably maintained. CD133^+^ cells were sorted from the cultures by anti-CD133/1-phycoerythrin and flow cytometry and used as cell sorce to re-initiate GSC culture lines. The GSC culture lines used in this study were derived from three glioblastoma tumors; D431 and S496 were derived from patients whom received radiation and chemotherapy prior to their recurrence and re-operation, and E445 was obtained prior to treatment. Based on prediction set of 595 gene hierarchical clustering by Freije et al., which subclassifies glioblastomas corresponding to four clinical relevant subtypes as categorized by The Cancer Genome Atlas (TCGA) Research Network [40], [41], the D431 tumor was assigned to MES subtype due to overexpression of extracellular matrix components and regulators. Meanwhile, S496 and E445 were assigned to PN subtype due to overexpression of genes involved in proliferation/mitosis and neurodevelopment, highlighting the poor prognosis and lack of therapies in all three cases.

### Isolation of treatment-resistant clonogenic clones and clonogenic survival assay

Dissociated GSC spheres were seeded in 60×15 mm gridded culture dishes on day 0 at 900 cells/dish in 2 ml stem cell culture media and incubated overnight. Cells were irradiated in 3 fractions of 4 Gy, at a dose rate of 0.7– 0.8 Gy per minute (Gulmay Medical) on days 1, 4, and 7. This radiation dose regimen is approximately 1/5 of the dose used in clinical treatment. For chemoradiation, 5 µM TMZ was added to cell cultures 2 hrs before the first dose of 4 Gy RT and after each RT fraction, while simultaneously replacing half of the medium with fresh medium (concurrent treatment). After a 2-day break, 10 µM TMZ was added for an additional 4 days after a 2-day interval (adjuvant treatment). After the course of fractionated irradiation with and without TMZ was completed, the surviving cell populations that formed colonies were counted on day 14 (Figure S1). Single colonies derived from non-treated plates and treated plates were picked up using a pipette, and individually expanded for further characterization.

### Proliferation assay for clonogenic clones

The proliferative activity of clonogenic clones were determined by a 3-(4, 5-dimethylthiazol-2-yl)-5-(3-carboxymethoxyphenyl)-2-(4-sulfophenyl)-2H-tetrazolium (MTS/PMS) colorimetric assay according to the manufacturer's instructions (Promega). Cells were incubated for 72 hours and the absorbance was measured at 490 nm.

### Cell cycle analysis of 5-bromo-2-deoxyuridine (BrdU) labeled cells

A BrdU pulse-chase time course was conducted for measuring cell turnover using APC BrdU Flow Kit (BD pharmingen). Briefly, cells were pulsed with BrdU (10 µM) for 1 hr. The cells were washed and incubated further at 37°C for 3 h and 6 h, after which the cells were harvested, fixed, and permeabilized with BD Cytoperm Permeabilization Buffer Plus. Cells were then incubated with DNase to expose incorporated BrdU, followed by addition of APC-conjugated anti-BrdU antibody and 7-amino-actinomycin (7-AAD). The cell cycle analysis of BrdU^+^ cells for each time point was acquired on a BD FACSVerse system.

### Tumor formation assay and histopathological analysis

The tumorigenicity of sensitive and resistant clones was determined by injecting 10^5^ live cells in a volume of 3 µl into NOD.CB17-Prkdcscid/J mice intracranially (i.c.). Mice were sacrificed if neurological symptoms started to show. Tumor tissues were collected and subjected to hematoxylin-eosin (H-E) staining for histopathological analyses. All animal work in this study was approved by UCLA Institutional Animal Research Committee (ARC # 2005-063-22).

### Microarray procedures, data analysis and gene annotation

Molecular profiling of sensitive and resistant GSC clones was performed using standard Affymetrix protocols and hybridized to Affymetrix GeneChip U133 Plus 2.0 Array as described previously [40]. The group comparisons were performed in dChip and samples were permuted 100 times to assess the false discovery rate (FDR). Probe set signals that were ≥2-fold in resistant versus sensitive group and with a pairwise t-test (P<0.05) were selected. All microarray CEL files analyzed in this study are accessible from the Gene Expression Omnibus (GEO) (Series Accession number: GSE46531). Functional annotation of individual gene was obtained from NCBI/Entrez Gene (http://www.ncbi.nlm.nih.gov/sites/entrez), Uniprot (http://www.uniprot.org/), information hyperlinked over protein (http://www.ihop-net.org/), neXtProt (http://www.nextprot.org/), BioGraph (http://biograph.be/about/welcome) and the published literature in PubMed Central (http://www.ncbi.nlm.nih.gov/pubmed).

### Semi-qt reverse transcriptase polymerase chain reaction (RT-PCR) analysis

Total RNA was extracted from cells using an RNeasy kit (QIAGEN). Two micrograms of RNA from each sample were transcribed to cDNA using a Taqman RT Reagent Kit (Applied Biosystems). PCR was performed, using 5 µl cDNA equivalents to 100 ng total RNA and was carried out by using SYBR Green PCR Core Reagents (Applied Biosystems). The reactions were cycled 30 times [50°C for 2 minutes and 95°C for 10 minutes (94°C for 15 seconds, 58–60°C for 1 minute, and 72°C for 1 minute) x 30 cycles]. PCR products (5 µl) were electrophoresed on 2% agarose gel. The primer sequences and expected size of PCR products are described in Table S1.

### siRNA transfection

A reverse transfection protocol was performed to deliver gene-targeted siRNA (Ambion) or non-silencing control siRNA (Ambion) into cells. Briefly, a transfection complex was prepared by diluting siRNA in 10 µl OPTI-MEMI (Invitrogen) then adding 10 µL OPTI-MEMI containing 0.3 µL Lipofectamine RNAiMAX transfection reagent (Invitrogen). This complex was then added into each well in a 96-well plate followed by seeding 6000 GSCs in 100 µL media to give a final siRNA concentration of 30 nM in each well. Targeted gene silencing was determined 72 hrs after transfection by qtRT-PCR, using Power SYBR® Green Cells-to-CT™ Kit (Ambion).

### Western blot analysis

20 µg protein from each sample were separated on 10% SDS-PAGE (Bio-Rad) and transferred onto a PVDF membrane. The blots were incubated with the primary antibodies overnight at 4°C. The following primary antibodies were used: Rabbit anti-human p53, phospho-p53 (Ser20), AMPKα, phospho-AMPKα (Thr172), Akt, phospho-Akt (Thr308) (all at 1∶1000), and β-actin (1∶2000, Cell Signaling). The blots were washed and incubated with horseradish peroxidase-conjugated anti-rabbit IgG for 1 h. Then, the blots were washed again, incubated with Pierce Supersignal ECL substrate, and exposed to X-ray films.

### Functional assays of cell metabolism

ATP levels were determined using a luciferin–luciferase-based bioluminescence assay (Molecular Probes® ATP Determination Kit, Invitrogen) according to the manufacturer's instructions. 10^4^ cells/well were plated in 96-well plates and exposure to 2-deoxyglucose (2-DG), histidine, or Etomoxir (all from Sigma) at the concentrations indicated in the figures and incubated for 45 minutes. Luminescent intensity was measured by a SpectraMax M5e Multi-Mode Microplate Reader (Molecular Devices).

2-(N-(7-nitrobenz-2-oxa-1, 3-diazol-4-yl) amino)-2-deoxyglucose, a fluorescently-labeled deoxyglucose analog (2-NBDG, Cayman Chemical), was used as a probe for detecting glucose uptake by cells. Cells were seeded at a concentration of 4×10^5^ cells/ml/well in a 6-well plate, in duplicate, and incubated overnight at 37°C. Cells were washed twice and incubated with 10 µM 2-NBDG in glucose-free culture media in the presence of 1 µM insulin for 20 min. Cells cultured in media without 2-NBDG were used as negative control. Cells were washed twice prior to flow cytometric analysis.

2′7′-dichlorofluorescein diacetate (DCF-DA) (Sigma) was used to detect reactive oxygen species (ROS) in cells. 4×10^5^ cells/ml/well in a 6-well plate were pretreated with or without 5 mM Tiron, an ROS scavenger (Sigma), at 37°C for 30 minutes. Cells were washed and incubated with 20 µM DCF-DA in PBS at 37°C for 30 min, then washed twice with PBS. Cells were resuspended with PBS containing 500 µM H_2_O_2_ and subjected to flow cytometric analysis.

Intracellular oxidized nicotinamide adenine dinucleotide (NAD^+^) were determined by NAD+/NADH cell-base assay kit (Cayman Chemical) according to the manufacturer's instructions. 10^4^ cells/well were plated in 96-well plate for overnight. Culture media was removed and cells were lysed. After centrifugation, 100 µl supernatant and titrated standards were transferred to a new plate followed by adding 100 µl reaction solution. Absorbance of each sample was measured using a microplate reader at a wavelength of 450 nm.

The endogenous levels of phosphorylated AMPK (pAMPK α/Thr172) and phosphorylated Akt (phospho-Akt/Thr308) in cells were determined by PathScan® Phospho-AMPKα and Phospho-Akt Sandwich ELISA Kits (Cell Signaling), respectively, according to the manufacturer's instructions. 70 µg protein from each sample was used in the assay. Absorbance of each sample was measured at a wavelength of 450 nm.

Autophagy was determined by LysoTracker® Red DND-99 (Invitrogen). The quantitation was based on a correlation between autophagic activity and overall lysosomal acidity. Cells were incubated with prewarmed fresh media containing 75 nM probes at 37°C for 1 hr. After incubation, the stained cells were washed and resuspended with 500 µl PBS, and analyzed by flow cytometric analysis.

### Exome sequencing

To detect DNA changes in coding regions at single base resolution and regional copy number changes, we performed exome capture of sensitive and resistant GSC clones, using the SureSelect Human All Exon Kit (Agilent). The samples were tagged with a unique barcode and sequenced using a paired-end protocol on a portion of a lane of an Illumina HiSEQ instrument, to generate an average of 100x base coverage over the exome of unique independent reads sufficient for high-quality diploid genome sequencing. Generated reads were aligned to human reference genome using Novoalign (www.novocraft.com), sorted, and stored in bam (binary SAM) format. Reads corresponding to PCR duplicates were marked with the Picard MarkDuplicates tool (picard.sf.net), and the Broad Institute's Genome Analysis Toolkit (GATK) was used for recalibrating base quality recalibration, indels realignment, and to call and annotate variants. Tools from GATK, along with custom scripts and tools, were used to select treatment-sensitive and treatment-resistant clone-unique mutations and to find commonly mutated sites, genes, and pathways among the different clones. Copy number variation (CNV) and loss of heterozygosity were analyzed by ExomeCNV.

### Statistical analysis

Each experiment was set up in duplicate or triplicate, and repeated at least twice. Data were expressed as means ± SD and analyzed using one-way ANOVA tests, depending on homogeneity of variances. All *P*-values were two-sided, and values lower than 0.05 were considered significant. SPSS v13.0 for Windows software was used for all statistical analyses.

## Results

### A minority of clonogenic cells in GSC cultures resist radiation and radiochemotherapy

**Figure 1:**
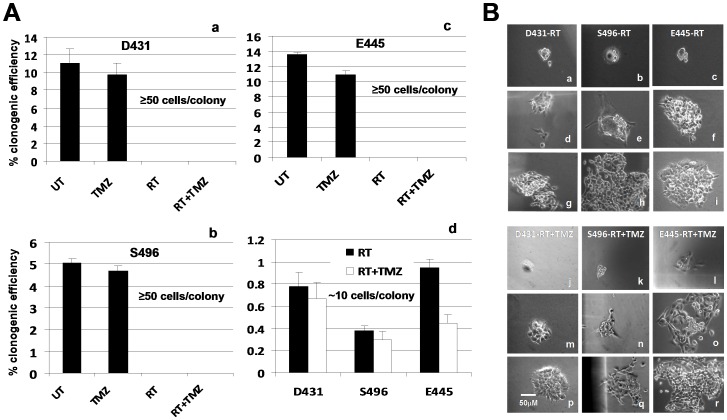
Cells repopulated from tumor-derived clonogenic cells are sensitive to RT or RT+TMZ treatment; only a few slow-growing clonogenic cells can survive and regain their self-renewal capacity. A. Dissociated cells from GSC cultures derived from three patients' tumors as indicated were plated in culture dishes at clonal density of 900 cells/2 ml/60 mm dish. Cells were incubated for overnight and subjected to treatment as described in Figure S1. Untreated cells (UT) or treated with TMZ alone (10 µM every 3 days) served as control. Colonies that contained at least 50 cells were counted on day 14 in all dishes (a-c) while small colonies that contained at least 6 cells were also counted in plates treated with RT and RT+TMZ (d). Data represent mean values ± SD of triplicate dishes. B. Light-microscopic morphology of clonogenic survivors after treatment. Few single cells survived treatment (a–c, j–l) (days 2–3) and slowly regained self-repopulating capacity (d–f, m–o) (days 14–21), (g–i, p–r) (days 25–30).

We have previously established and characterized several patient tumor-derived CD133^+^ GSC culture lines [7], [42]. The CD133^+^ GSC cells purified from tumor sphere cultures express both radial glial and neural crest cell developmental genes and are capable of clonal self-renewal and division to produce CD133^−^ fast-growing progeny that are morphologically heterogeneous revealed by differences in cell sizes and shapes, which form the major cell population within tumor spheres [7]. We found that long-term passaged GSC lines typically contain 3-30% CD133^+^ cells even initiated by purified CD133^+^ cells, and suggested that slow self-renewal and fast proliferative division/differentiation naturally occur during passaging in serum-free media containing stem cell growth factors [7]. In our hands, besides purified CD133^+^ GSC from tumor sphere cultures, most clonogenic cells repopulated from CD133^+^ GSC cultures (e.g. single cell-derived colonies/spheres) could reconstitute a tumor in mouse brain. Moreover, the tumor xenograft initiated by these single-cell derived tumor spheres/colonies could be maintained by serial passage in the animals, suggesting patient tumor-derived CD133^+^ tumor sphere cultures may contain heterogeneous population of stem-like cells with tumor-initiating capacity. To understand the mechanisms underlying glioblastoma resistance to RT or RT+TMZ, we isolated treatment-resistant clonogenic cells from three established CD133^+^ GSC cultures [7], [42] by treating them with fractionated RT (4 Gy/fraction, 3 fractions per week for 1 wk) or concomitant RT plus TMZ (5 µM) followed by adjuvant TMZ (10 µM) as described in Materials and Methods and Figure S1. The clonogenic survival assays showed that untreated (or pretreated) cultures seeded with 900 cells (derived from GSC cultures) per 60 mm diameter dish exhibited 5-14% clonogenic efficiency (CE) when colonies of more than 50 cells were counted on day 14, whereas RT with or without TMZ treatment caused a massive killing of cells within two to three fractions, and no colonies with more than 50 cells were counted (Figure 1A, a–c). However, we have observed a few surviving single cells which have slowly grown into colonies (all had fewer than 20 cells per colony on day 14 with a survival rate of 0.3-0.95%) (Figure 1A, d). Cultures treated with RT+TMZ had a lower CE than those treated with RT alone. These clonogenic survivors gradually grew into larger colonies (Figure 1B) and were picked and expanded individually, and designated as treatment-resistant GSC clones or resistant clones. In parallel, selected large colonies (>50 cells/colony) were picked up from the untreated plates on day 14 after seeding and were designated as treatment-sensitive GSC clones or sensitive clones. Cells treated with 10 µM TMZ alone showed similar CE to that of the untreated cells and were not recruited for a follow-up study.

### Treatment-resistant GSC clones gave rise to both resistant and sensitive clones and repeated treatment further promotes their enrichment

**Figure 2:**
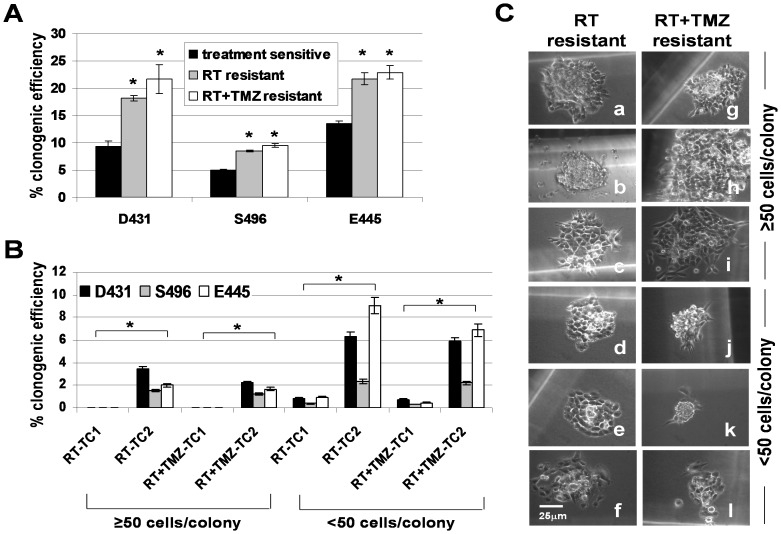
Cells expanded from treatment-resistant GSC clones contain more clonogenic cells than those of treatment–sensitive GSC clones and repeated treatment further promoted the expansion of resistant clones. A. The clonogenic efficiency was determined in cells populated by resistant and sensitive clones, using limiting dilutions. Data represent mean values ± SD of triplicate wells. *p<0.05 versus sensitive clones. B. Clonogenic efficiency was determined in resistant clone-derived cells which underwent a second treatment cycle (TC2). Colony counts were performed on day 14. Data represent mean values ± SD of triplicate dishes. *p<0.05 versus treatment cycle 1 (TC1). C. Light-microscopic morphology of colonies repopulated by resistant clones recovered from a second treatment cycle of RT (a–f) (day 14) and RT+TMZ (g–l) (day 14).

Next, we examined whether treatment-selected, single cell-derived resistant clones were more clonogenic than single-cell-derived sensitive clones and whether repetitive treatment results in further enrichment of resistant clones. The clonogenic assay showed that the cells reseeded by resistant clones contained significantly higher numbers of clonogenic cells than those repopulated from sensitive clones (Figure 2A). Clonogenic cells grown from resistant clones however, were still sensitive to a second cycle of RT treatment (TC2); but, more cells survived and unlike in the first cycle (TC1), formed colonies of >50 cells at day 14 with a clonogenic survival rate (CSR) of 1.2-3% (Figure 2B, 2C). Moreover, the number of small colonies (10–50 cells/colony) were significantly increased (6–15 fold) (Figure 2B, 2C) after the second treatment cycle that gradually grew into large colonies within 3–4 weeks. These data thus indicate that although RT or RT+TMZ treatment could still deplete the majority of cell population grown from resistant clones, treatment also simultaneously selected for a small number of resistant clones, while further treatment cycles can increasingly expand them. These observations also demonstrated a hierarchy of self-renewing resistant clones in the tumor sphere cultures capable of generating heterogeneous population containing resistant clones, sensitive clones, and non-clonogenic cells (Figure S2). The cellular diversity in resistant clone-derived populations apparently exhibited differential sensitivity to the treatment.

### Treatment-resistant GSC clones are slow-cycling, stem-cell like, tumor-initiating cells

**Figure 3:**
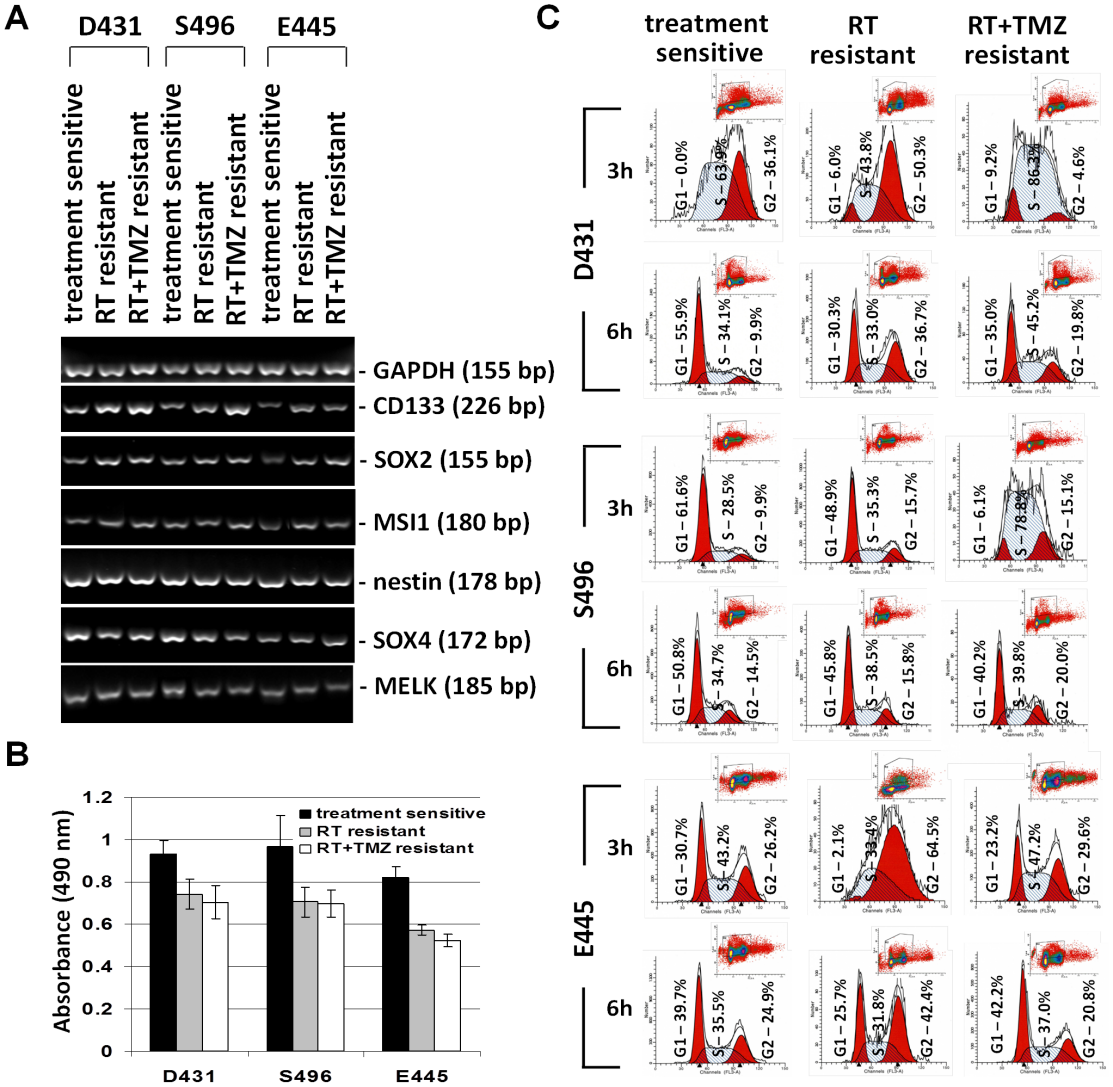
Treatment-resistant GSC clones are slow-cycling cells express upregulated stem cell markers. A. Total RNA of sensitive clones and resistant clones were extracted. The mRNA expression levels of indicated stem cell-associated genes were analyzed by semi-qtRT-PCR with specific primers. β-actin was used as an internal control gene. B. Proliferative activity of cells populated by resistant clones and sensitive clones was determined by 3-day MTS/PMS cell proliferation assays. Data represent mean values ± SD of triplicate measurements. C. Sensitive clones and resistant clones were pulsed with 5-bromo-2-deoxyuridine (BrdU) for 1 hr, and cell cycle of BrdU^+^ cells was analyzed 3 hrs and 6 hrs after BrdU pulsing. Cell cycle phases were defined by 7-amino-actinomycin (7-AAD) staining intensities.

Since both sensitive and resistant clones are clonogenic and were selected from tumorigenic bulk GSC cultures, we tested whether treatment resistance is associated with expression of stem cell markers. The qtRT-PCR analysis revealed that mRNA levels of CD133, SRY (sex determining region Y)-box 2 (SOX2) and musashi homolog 1 (MSI1) (except S496-RT) were upregulated in resistant clones when compared to sensitive clones, but not nestin, SOX4, or maternal embryonic leucine zipper kinase (MELK) (Figure 3A).

Correspondingly, resistant clones exhibited slightly less proliferative activity in culture as determined by short-term proliferation assays (72 hrs) (Figure 3B). To determine whether resistant clones are indeed slow-cycling cells when compared to sensitive clones, we performed a BrdU pulse-chase time course at 3-hr and 6-hr time points for measuring progression of BrdU-labeled cells through the cell cycle (Figure 3C). The data indicated that for the D431 cells at the 6-hr time point, 55.9% cells in sensitive clones, 30.3% cells in RT-resistant clones, and 35.0% cells in RT+TMZ-resistant clones have completed a cell cycle. For the S496 cells at 3-hr time point, 61.6% cells in sensitive clones, 48.9% cells in RT-resistant clones, and 6.1% cells in RT+TMZ-resistant clones have completed a cell cycle. For the E445 cells at 3-hr time point, 30.7% cells in sensitive clones, 2.1% cells in RT-resistant clones, and 23.2% cells in RT+TMZ-resistant clones have completed a cell cycle. Thus, resistant clones are slow-growing when compared to sensitive clones.

**Figure 4:**
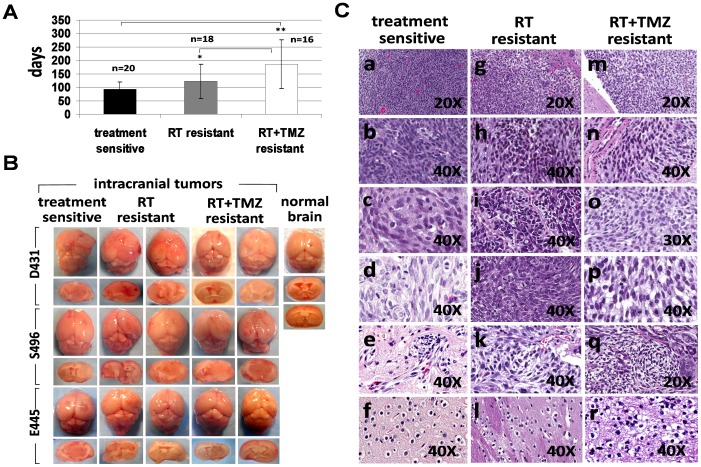
Treatment-resistant GSC clones exhibited a delay in tumor formation compared to those of treatment-sensitive GSC clones. A. 2×10^5^ cells derived from treatment-sensitive clones, RT-resistant clones, and RT+TMZ-resistant clones were stereotactically injected into the brains of SCID mice and days required for developing neurological signs by tumor growth in each mouse were recorded. Data represent mean values ± SD of indicated numbers of animals that have developed tumors. *p<0.05 versus RT+TMZ-resistant clones, **p<0.001 versus treatment-sensitive clones. B. Representative macrophotographic image of glioma xenografts initiated by treatment-sensitive clones, RT-resistant clones and RT+TMZ resistant clones that are growing in intracranial site. C. Representative hematoxylin and eosin (HE) staining of xenograft tumors. Brain tissues from mice injected with either treatment-sensitive or treatment-resistant clones display invasive growth of gliomas and exhibits histopathological features of human glioblastoma, including hypercellularity (Figure 4C, a, g, m), hyperchromatism (Figure 4C, b, h, n), pleomorphism (Figure 4C, c, i, o), mitosis (Figure 4C, d, j, p), vascular endothelial hyperplasia (Figure 4C, e, k, q), and oligodendroglial components (Figure 4C, f, l, r). Magnification, 20X and 40X as indicated.

To test whether treatment-sensitive and treatment-resistant clones possess tumorigenic capacity, 2×10^5^ cells derived from sensitive clones and resistant clones were stereotactically injected into the brains of SCID mice. Mice which received cells derived from sensitive clones (18/18), RT-resistant clones (17/18), or RT+TMZ-resistant clones (16/18) all developed tumors, but the growth of RT+TMZ-resistant clones was significantly delayed when compared to that of sensitive clones (p = 0.0015) or RT-resistant clones (p = 0.0376) (Figures 4A, 4B). Although tumor growth from RT-resistant clones was delayed compared to tumor growth from sensitive clones, the delay did not reach statistical significance (p = 0.0659). The H-E staining of xenograft tumors initiated by sensitive or resistant clones all demonstrated invasive growth of gliomas with diffuse infiltration into the surrounding tissue and vessels, and recapitulated the histopathological features of human glioblastoma (Figure 4C, a-r). Clonogenic tumor cells cultured from xenografts initiated by resistant clones can reinitiate tumors in new hosts in a series of transplants (data not shown), confirming the characteristics of tumor stem cells in resistant clones.

### Molecular signatures reveal defensive strategies of treatment-resistant GSC clones are mainly associated with responses of reduction of glucose usage and activation of cellular and genomic maintenance and repair pathways

To explore the differential molecular properties allowing resistant clones to overcome or adapt to deadly radiochemotherapy, we performed a comparative high-density microarray analysis. We first determined the common genes that are overexpressed in RT-resistant clones and RT+TMZ-resistant clones when compared to group of sensitive clones. Six sensitive clones (n = 3 patients, 2 clones per patient), 3 RT-resistant clones (n = 3) and 3 RT+TMZ-resistant clones (n = 3) were used for the analysis. Fifty-three informative genes were eluted with a FDR of <0.1%, segregating the 2 groups (Table 1). Notably, many of the identified genes are involved in multiple functions or serve similar functions. The overall gene expression profile revealed a complex of interconnected defense strategies highlighting the blocking of IIS, decreased glucose uptake, augmentation of lipid catabolism, activation of oxidative stress responses, suppression of growth, differentiation and inflammation, stimulation of angiogenesis, migration, anti-apoptosis, and amplification of cellular and DNA maintenance and repair activities.

**Table 1:** Molecular signatures and defense profiles of treatment-resistant glioblastoma stem cell clones.

Gene Name and Gene Symbol	Fold Change	P-Value	Functional Involvement
MALAT1: metastasis associated lung adenocarcinoma transcript 1	14.38	0.002085	antiapoptosis; migration; invasion; metastasis
ENPP2: ectonucleotide pyrophosphatase 2	10.98	0.019869	insulin resistance; lysosome biogenesis; antiapoptosis; cell migration; angiogenesis
TXNIP: thioredoxin interacting protein	8.92	0.049541	blocking glucose uptake; oxidative stress; antigrowth
SUPT16H: suppressor of Ty 16 homolog (S. cerevisiae)	6.75	0.000119	histone chaperon; DDR; checkpoint activation; DSB repair; transcription
EGR1: early growth response 1	6.29	0.020384	impaired insulin/akt signaling; reduced glucose uptake; induction of sirt1 expression; autophagy
SSFA2: sperm specific antigen 2	6.22	0.008552	reduce glucose uptake; reduce metabolic rate; structural integrity
COL6A2: collagen, type VI, alpha 2	5.52	0.009504	antiapoptosis; cell migration and adhesion
IL6ST: interleukin 6 signal transducer (gp130)	5.46	0.000039	cell migration; antiapoptosis; suppress insulin/akt signaling; angiogenesis
EPRS: glutamyl-prolyl-tRNA synthetase	5.42	0.000664	translational control of inflammatory genes
MFAP4: microfibrillar-associated protein 4	5.34	0.027521	prevention of ECM degradation and aggravated elasticity
VEGFA: vascular endothelial growth factor A	5.30	0.011344	angiogenesis; vasculogenesis; endothelial cell growth and migration
NFIX: nuclear factor I/X	5.14	0.006796	astrogenesis/gliogenesis; adhesion, migration and invasion
ALDH3A2: aldehyde dehydrogenase 3 family, member A2	5.01	0.011524	detoxification of lipid peroxidation product; suppress ER stress; oxidizes aldehydes to fatty acid
FBN1: fibrillin 1	4.52	0.028724	structural support in microfibrils that form elastic fibers
ZC3H11A: zinc finger CCCH-type containing 11A	4.48	0.000062	phosphorylated upon DNA damage recognized byATM and ATR
C5orf24: chromosome 5 open reading frame 24	4.43	0.000421	phosphorylated upon DNA damage recognized byATM and ATR
PLD3: phospholipase D family, member 3	4.33	0.004337	lipid catabolism; block insulin/Akt signaling; oxidative stress; cell survival
FMNL2: formin-like 2	4.33	0.035379	epithelial-mesenchymal transition; cell motility and invasion
TPR: translocated promoter region (to activated MET)	4.10	0.013417	DSB repair; telomere maintenance; recruitment of spindle checkpoints
PPFIBP1: PTPRF interacting protein, binding protein 1	4.01	0.009418	maintain lymphatic vessel integrity; cell adhesion, migration, invasion
RPS11: Ribosomal protein S11	3.99	0.000005	Antiapoptosis; selecting the correct tRNA in protein biosynthesis
TPM4: tropomyosin 4	3.96	0.000159	stabilizing, repair and regeneration of cytoskeleton actin filaments
SLC38A1: solute carrier family 38, member 1	3.85	0.000536	glutamine transporte; oxidative stress; detoxification
RPS20: ribosomal protein S20	3.83	0.012844	stabilize the folded structure of the ribosomal RNA
PSAT1: phosphoserine aminotransferase 1	3.83	0.000205	serine synthesis pathway; amino acid, phospholipid, and nucleotide synthesis
LOX: lysyl oxidase	3.81	0.018453	crosslinking of collagens and elastin; tumor suppression
ACTR2: ARP2 actin-related protein 2 homolog (yeast)	3.74	0.005364	cell migration; cell polarity maintenance; asymmetric cell division
DHX9: DEAH (Asp-Glu-Ala-His) box polypeptide 9	3.73	0.008356	DSB repair
HSP90B1: heat shock protein 90kDa beta (Grp94), member 1	3.61	0.001383	ER-associated protein degradation; unfolded protein response
NBPF family: neuroblastoma breakpoint family, members	3.57	0.000342	tumor suppressors linked to neuroblastoma
ATP13A3: ATPase type 13A3	3.56	0.002276	neuronal development; tumor suppressor
PPP1R3C: protein phosphatase 1, regulatory subunit 3C	3.55	0.019646	suppress glycogen breakdown; glycogen accumulation
FAM114A1: family with sequence similarity 114, member A1	3.54	0.020270	neuronal cell development
MATR3: matrin 3	3.50	0.001110	ATM target; DSB response; DSB repair; antiapoptosis
RASSF8: Ras association(RalGDS/AF-6) domain family 8	3.47	0.000865	adherens junction function; cell migration; tumor suppressor
UBA6: ubiquitin-like modifier activating enzyme 6	3.37	0.000331	activates ubiquitin and FAT10; proteasomal degradation
C6orf62: chromosome 6 open reading frame 62	3.29	0.002501	uncharacterized
PDLIM5: PDZ and LIM domain 5	3.16	0.001477	cytoskeleton organization; antiproliferation; heart development
RPL38: ribosomal protein L38	3.13	0.000001	translational control of Hox gene expression; tissue patterning; antidifferentiation; antidevelopment
PRRG4: Proline rich Gla (G-carboxyglutamic acid) 4	3.08	0.012058	downregulates ERK ½ signaling; cell cycle control
MAP4: microtubule-associated protein 4	2.90	0.012801	stabilizes mitochondria, microtubule network, and viability
PRKCI: protein kinase C, iota	2.89	0.000997	antiapoptosis; survival; microtubule dynamics
RAB2A: RAB2A, member RAS oncogene family	2.88	0.004642	microtubule dynamics
SF3B1: splicing factor 3b, subunit 1,155kDa	2.87	0.001173	cell spliceosome; repression of Hox genes, antidifferentiation; antidevelopment
RPL27A: Ribosomal protein L27a	2.77	0.000029	developmental patterning
HNRPA3: heterogeneous nuclear ribonucleoprotein A3	2.71	0.000016	stable maintenance of telomere repeats
BAT2D1: BAT2 domain containing 1	2.64	0.002660	cell cycle regulation
OSBPL8: oxysterol binding protein-like 8	2.48	0.000134	lipid receptors; modulate lipid homeostasis; suppress cholesterol synthesis
MOBKL1B: MOB1, Mps One Binder kinase activator-like 1B	2.45	0.000767	growth control; tumor suppressor; antiapoptosis
RAD23A: RAD23 homolog A (S. cerevisiae)	2.41	0.000113	nucleotide excision repair; proteasomal degradation; mitochondrial biogenesis
PPP2R1A: protein phosphatase 2, regulatory subunit A, alpha	2.36	0.000030	DSB repair; impairs insulin action/glucose metabolism/Akt activity; anti-growth; antiapoptosis
AGGF1: angiogenic factor with G patch and FHA domains 1	2.32	0.000417	Angiogenesis; vasculogenesis
FOLR1: folate receptor 1 (adult)	2.28	0.000301	DNA methylation; nucleotide synthesis; mitochondrial DNA stability; DNA repair; regeneration of CNS

NOTE: Probe set signals on the expression array that were ≥2-fold increased in relative expression in treatment-resistant glioblastoma stem cell (GSC) clones (n =  6 clones from 3 patients; 3 RT-resistant clones and 3 RT+TMZ-resistant clones) compared with treatment-sensitive GSC clones (n = 6 clones from 3 patients) were selected. Samples were permutated 100 times by dChip and identified 53 genes at false discovery rate (FDR) of <0.1%. DSB = double-strand break; ER = endoplasmic reticulum; ECM = extracellular matrix; DDR = DNA damage response; ATM = Ataxia telangiectasia mutated; ATR =  Ataxia telangiectasia and Rad3 related; CNS = central nervous system.

Evidently, a series of genes functioning in suppressing glucose uptake, inhibiting insulin/Akt signalling, and limiting glycogen breakdown (ENPP2, TXNIP, EGR1, SSFA2, IL-6ST, PLD3, PPP2R1A, PPP1R3C) were upregulated in resistant clones and suggest resistant clones exhibit a GR- and insulin-resistant-like phenotype. The upregulation of genes with functions associated with autophagy, lipid catabolism, and detoxification of lipid peroxidation products (ENPP2, ALDH3A2, PLD3, OSBPL8) further imply that resistant clones may use FAO as a major anaplerotic input to keep the tricarboxylic acid (TCA) cycle functioning for energy when the glycolysis pathway is limited. The overexpression of these genes also serves as an indication of the oxidative stress response of cells (TXNIP, EGR1, PLD3, SLC38A1), especially TXNIP, which is not only a potent negative regulator of glucose uptake and utilization, but also binds and inhibits thioredoxin, and thereby can induce oxidative stress.

As anticipated, molecular signatures of resistant clones were highly enriched with genes that promote genomic stability and cellular/cytoskeletal integrity. These genes address DNA damage response (DDR) (SUPT16H, ZC3H11A, C5orf24, MATR3), activation of cell-cycle/spindle checkpoints (SUPT16H, TPR), double-strand break (DSB) repair (SUPT16H, TPR, DHX9, MATR3, PPP2R1A, FOLR1), nucleotide excision repair (RAD23A), maintenance of telomere repeats (TPR, HNRPA3), stabilization of the folded structure of the ribosomal RNA (RPS20), the collagen fibrils and elastin integrity (MFAP4, FBN1, LOX), and maintenance of cytoskeleton organization and mitochondrial function (TPM4, PDLIM5, MAP4, PRKCI, RAB2A, RASSF8). Moreover, activation of genes associated with endoplasmic reticulum (ER)-associated unfolded protein response (HSP90B1), lysosome biogenesis (ENPP2), the ubiquitin-proteasomal pathways (UPP) (UBA6, RAD23A) and autophagic-lysosomal system (ALP) in resistant clones support the view that these cells are reacting to stress. The gene profiles also portrayed resistant clones an anti-apoptotic (MALAT1, ENPP2, COL6A2, IL6ST, MATR3, RPS11, PRKCI, MOBKL1B, PPP2R1A), anti-inflammatory (EPRS), migratory (MALAT1, ENPP2, COL6A2, IL6ST, VEGFA, NFIX, FMNL2, PPFIBP1, ACTR2, RASSF8), and angiogenic phenotypes (ENPP2, IL6ST, VEGFA, AGGF1). Their quiescent nature is evidenced by upregulation of a series of genes with a role in anti-growth, tumor suppression, anti-development, and anti-differentiation (TXNIP, LOX, NBPF, ATP13A3, RASSF8, PDLIM5, PRRG4, BAT2D1, MOBKL1B, PPP2R1A, RPL38, SF3B1). The differential gene expression in sensitive and resistant clones was confirmed by qtRT-PCR analysis (Figure S3). Similar molecular signatures were also detected when the comparison was performed against RT-resistant clones or RT+TMZ-resistant clones respectively (Table S2, Table S3), implicating they may use similar pathways to achieve their protective phenotype.

**Figure 5:**
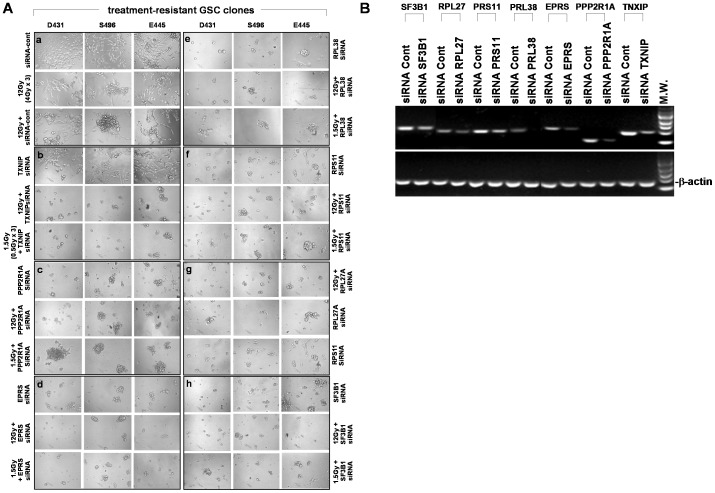
Sensitization of radiation treatment by knockdown of selected molecular signatures of treatment-resistant GSC clones. A. Cells derived from treatment-resistant GSC clones were treated with RT (12 Gy or 1.5 Gy) in the presence or absence of indicated siRNA targeting selected signatures of resistant clones. Photos were taken 7 days after treatment. B. Verification of on-target gene knockdown by indicated siRNA treatment. Total RNA from the resistant clones treated with specific siRNA was extracted. The mRNA expression levels of indicated genes were analyzed by qtRT-PCR with specific primers. β-actin was used as an internal control gene.

In order to test whether these upregulated genes have defensive functions, which contribute to the protective properties of resistant GSC clones, cells grown from resistant clones were pretreated with siRNA targeting selected signatures prior to receiving RT or RT+TMZ. As anticipated, cells derived from resistant clones treated with siRNA negative control with or without RT (4Gy x 3) all regain grow activity, while on-target knockdown of a series of selected genes associated with metabolic transformation and stress responses combined with RT treatment have resulted in the loss of cellular integrity (Figure 5A, 5B). Notably, effective treatment could also be achieved when combined with lower dose RT (0.5Gy x 3), indicating resistant clones have lost protective properties against treatment. Moreover, knockdown of selected genes alone without RT could also induce cell death and suggest that some of the molecular signatures may not only serve as defensive properties, but also serve as essential factors for maintaining cell survival (Figure 5A). Similar results were obtained when treatment was combined with RT+TMZ (data not shown).

### Treatment-resistant GSC clones use FAO pathway to maintain intracellular ATP levels and exhibit higher levels of oxidative stress compared to treatment-sensitive GSC clones

**Figure 6:**
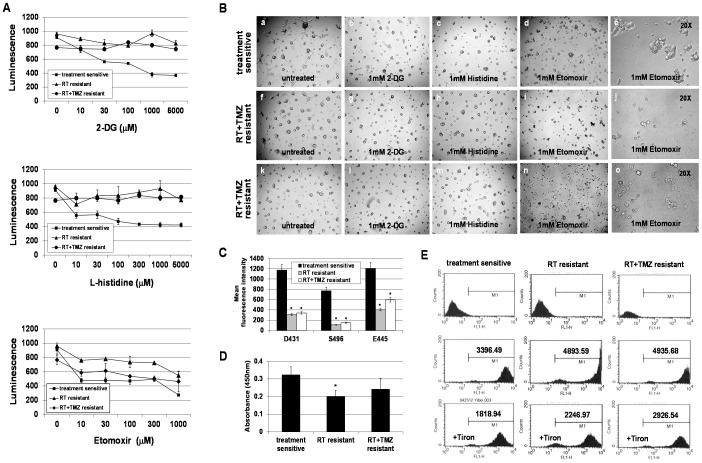
Treatment-resistant GSC clones use fatty acid oxidation (FAO), not glycolysis, as an essential energy source for maintaining intracellular ATP levels. A. Intracellular ATP levels of treatment-resistant clones and treatment-sensitive clones were measured using a luciferin–luciferase-based bioluminescence assay. Prior to assay, cells were treated with a glycolytic inhibitor (2-DG), a mitochondrial glutamine transport inhibitor (L-histidine), and a FAO inhibitor (Etomoxir), respectively, in a dose-escalating fashion as indicated. Cells without treatment served as controls. Luminescent intensity was measured by a luminescence microplate reader. B. Microscopic morphology of representative sensitive clones and resistant clones treated with indicated inhibitor. Magnification, 5X (a–d, f–i, k–n) and 20X (e, j, o). C. Glucose uptake by resistant clones and sensitive clones was measured by exposing cells to a fluorescently-labeled deoxyglucose analog (2-NBDG) in the presence of insulin. Mean fluorescence intensity was determined by flow cytometric analysis. Data represent mean values ± SD of triplicate dishes. *p<0.05 versus sensitive clones. D. Endogenous levels of phosphorylated of Akt at Thr308 was detected by a Phospho-Akt (Thr308) ELISA Kit. Seventy micrograms of cell lysates from each sample was used in the assay. The magnitude of the absorbance for the developed color is proportional to the quantity of Akt phosphorylated at Thr308. Data represent mean values ± SD of 3 clones, which are derived from 3 patients, in triplicate wells. *p<0.05 versus sensitive clones. E. Reactive oxygen species (ROS) generation by sensitive clones and resistant clones was measured using dichlorofluorescein diacetate (DCF-DA), which transforms to fluorescence by interacting with oxidants. Prior to measurement, cells were treated with or without 5 mM Tiron. ROS production was assayed by flow cytometric analysis.

Based on the molecular signatures of resistant clones, we hypothesized that resistant clones are in a “GR” status and lipid catabolism has become a major energy source. To test this hypothesis, we determined the intracellular ATP production in resistant clones in the presence of 1) 2-DG, an inhibitor of glucose uptake and glycolysis, 2) L-histidine, an inhibitor of mitochondrial glutamine transport, or 3) Etomoxir, an inhibitor of mitochondrial carnitine palmitoyltransferase I (CPT-1) which blocks FAO, and compared results with those of sensitive clones in the same condition. Blocking glucose uptake or glutamine transport partially decreased ATP production in only sensitive clones, not resistant clones, whereas inhibiting the FAO pathway altered ATP levels in both sensitive and resistant clones (Figure 6A). Altered ATP levels in sensitive clones led to reduced cell growth activity and colony size as compared to those without treatment (Figure 6B). In contrast, major cell death was only observed in Etomoxir-treated resistant clones while no obvious effects were seen in the other two conditions (Figure 6B). The glucose uptake assay further confirmed reduced glucose uptake by resistant clones under insulin stimulation (Figure 6C). This reduced glucose usage is not likely mediated by long-term culturing in glucose/insulin-containing media since both sensitive and resistant clones were cultured under the same condition and passages. Moreover, attenuated AKT activity was determined in resistant clones when compared to that of sensitive clones (Figure 6D), indicating resistant clones are less dependent on glucose, and that FAO becomes a crucial bioenergetic pathway for maintaining them. These data also suggest a close link between lipid catabolism and stress-resistant phenotype. Since resistant clones seem to favor FAO metabolism as the main energy source, we hypothesized that resistant clones may have higher levels of oxidative stress compared to sensitive clones. Indeed, increased ROS production by resistant clones was determined when compared to that of sensitive clones (Figure 6E). These data therefore support the notion that increased oxidative stress and fatty acid-supported mitochondrial respiration may promote and maintain the stress-resistant phenotype of resistant clones.

### Upregulation of SIRT1-AMPK signaling, autophagic activity, and global DNA repair transcripts in treatment-resistant clones

**Figure 7:**
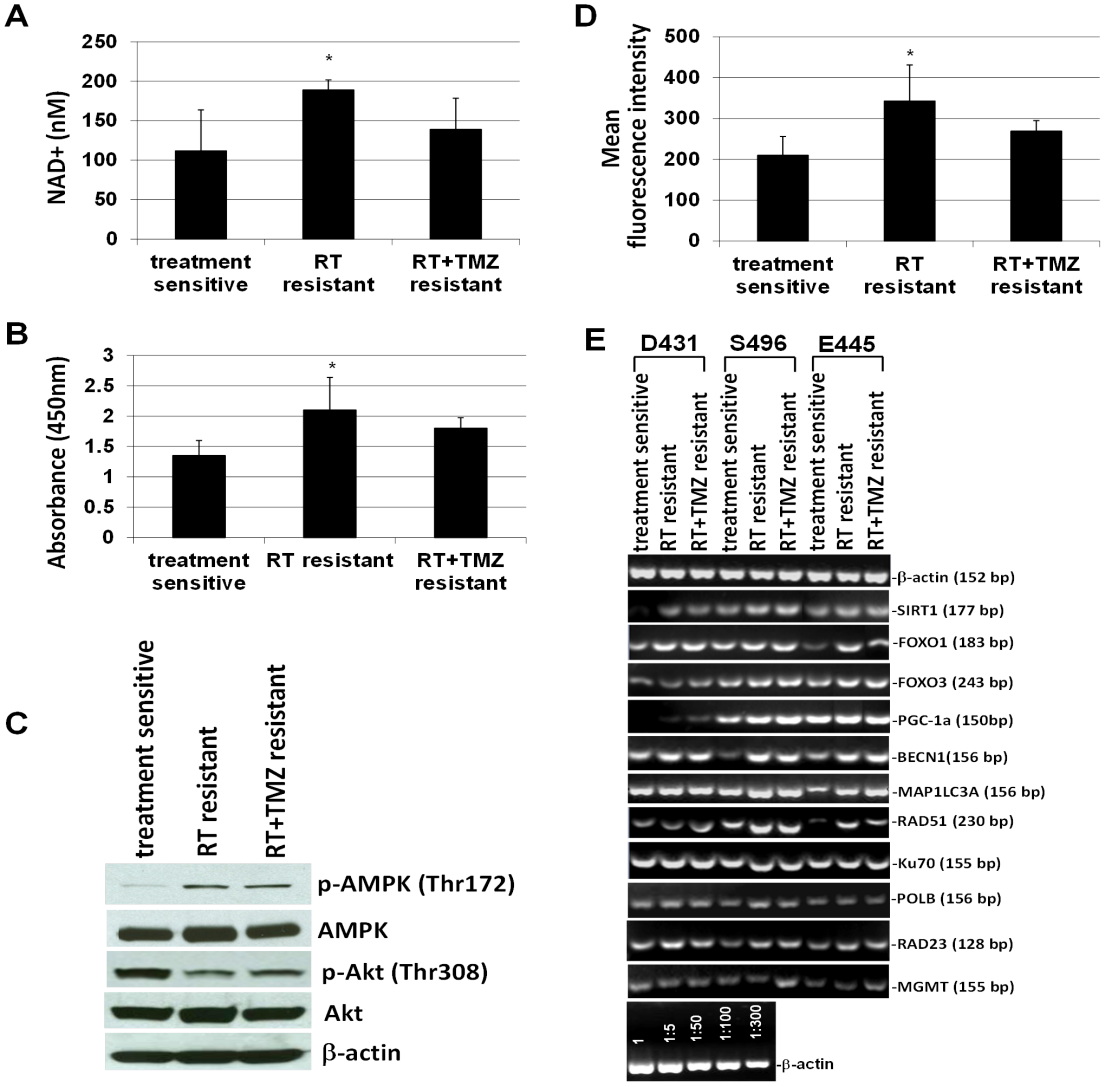
Regulation of cellular and molecular activities associated with metabolic adaptation to reduced glucose usage in treatment-resistant GSC clones. A. Intracellular NAD^+^ levels in sensitive clones and resistant clones (10^4^ cells/well) were determined by a NAD^+^/NADH colorimetric assay kit. The amount of NAD^+^ in cell lysate was quantified by comparing with NAD^+^/NADH standard solutions. B. Endogenous levels of pAMPK in resistant clones and sensitive clones were determined by a phospho-AMPKα (Thr172) ELISA Kit. Seventy micrograms of cell lysates from each sample were used in the assay. The magnitude of the absorbance for the developed color is proportional to the quantity of AMPKα phosphorylated at Thr172. C. Representative image of Western blot analysis of AMPKα, phospho-AMPKα (Thr172), Akt, phospho-Akt (Thr308), or β-actin expressed in sensitive clones and resistant clones. D. GSC autophagy is measured with a fluorescent acidotropic dye and flow cytometry based on a correlation between autophagic activity and overall lysosomal acidity. The intensity of lysosomal staining is proportional to lysosomal acidity. Data in A, B, and D represent mean values ± SD of 3 clones, which are derived from 3 patients, in triplicate wells. *p<0.05 versus sensitive clones. E.The mRNA expression levels of indicated genes were analyzed by semi-qtRT-PCR with specific primers. β-actin was used as an internal control gene.

It is well-documented that deacetylase SIRT1 is required for the induction of a “longevity phenotype” by GR/CR [34], [35], and it has been suggested that CR activates AMPK, a metabolic fuel gauge, which enhances SIRT1 activity by increasing cellular NAD^+^ levels, resulting in the deacetylation of downstream SIRT1 targets that include PGC-1α, forkhead box O1 (FOXO1) and FOXO3 [43]. Moreover, CR promotes cell survival via deacetylation of DNA repair factor Ku70 by SIRT1 [44], whereas loss of SIRT1 impairs DNA damage response and reduces the ability to repair DNA damage [45]. Likewise, autophagy is an essential part of the anti-aging mechanism of CR [33]. It is a catabolic process responsible for degrading damaged organelles and protein aggregates via lysosomal degradation machinery and recycling long-lived macromolecules for maintaining energy production during nutrient stress. As anticipated, levels of NAD^+^ and pAMPKα were higher in resistant clones compared to those in sensitive clones (Figure 7A, 7B). However only the increase in RT-resistant clones reached statistical significance. The upregulation of pAMPKα expression in resistant clones was also demonstrated by Western blot analysis, and that was accompanied by the downregulation of pAkt when compared to sensitive clones (Figure 7C). Higher autophagic activity was also detected in resistant clones compared to sensitive clones (Figure 7D), while activity was most enhanced in RT-resistant clones. Semi-qtRT-PCR analysis revealed that most resistant clones have slightly increased transcriptional levels of SIRT1 and its downstream targets, PGC-1α, FOXO1, and FOXO3 transcription factors when compared to autologous sensitive clones. Likewise, higher transcriptional levels of Beclin-1 (BECN1) and microtubule-associated protein 1 light chain 3 alpha (MAP1LC3A), two genes which play a central role in autophagy, were also determined in most resistant clones (Figure 7E). Correspondingly, transcriptional levels of RAD51, Ku70, polymerase-β (POLB), and RAD23A (detected by expression microarray), four genes that are known to be involved in the repair of DSBs by homologous recombination (HR), the repair of non-homologous end joining (NHEJ), base excision repair (BER), and nucleotide excision repair (NER), were all found to show some degree of upregulation in most resistant clones when compared to autologous sensitive clones. Notably, among the 4 DNA repair genes, RAD51 showed the highest extent of upregulation in resistant clones (Figure 7E). Meanwhile, all tested GSC clones expressed MGMT while only 2 out of 3 RT+TMZ-resistant clones expressed upregulated MGMT when compared to that of autologous sensitive clones (Figure 7E).

### Molecular subclassification and genetic changes in treatment-resistant GSC clones

**Figure 8:**
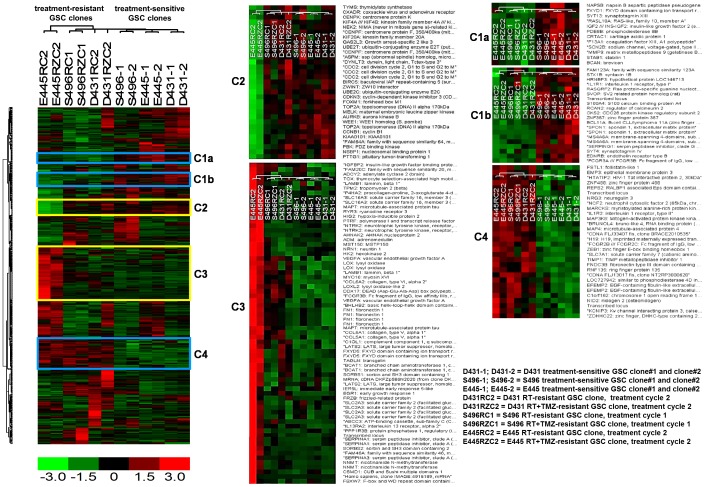
Molecular subclassification of treatment-sensitive and treatment-resistant GSC clones. Non-supervised hierarchical clustering of sensitive clones and resistant clones with prediction set of 595 genes. The heatmap with dendrogram showed hierarchical biclustering of genes differentially expressing mesenchymal subtype-associated genes and proneural subtype-associated genes which segregated treatment-sensitive and resistant GSC clones of E445 (C3) from all the others (C2). Genes distinctly expressed between sensitive clones and resistant clones were also identified (C1a, C1b, and C4).

Recent genomewide transcriptome analyses suggest that tumor heterogeneity of glioblastoma tumors categorized into four clinical subtypes also contain distinct GSC subtypes [46]. Since resistant clones were selected via RT or RT+TMZ, it is possible that radiation therapy may introduce genetic changes that contribute a cause to explain the distinct properties in resistant clones. To clarify this possibility, we performed an unsupervised sample clustering using Freije et al. predictive 595 gene list [40], which would allow for determining whether resistant clones maintain glioma properties as well as which subtype they fall under. The hierarchical clustering dendogram of sensitive and resistant clones with prediction set of 595 genes showed hierarchical biclustering of genes differentially expressed MES and PN subtype-associated genes (C2 and C3) that segregated mesenchymal E445-RT/E445-RT+TMZ from all the others that express PN-associated genes (Figure 8). It is interesting to note that untreated E445 sensitive clones was clustered as PN subtype distinct from autologous E445 resistant clones whereas that D431-resistant clones/D431-sensitive clone were originated from MES tumor subtype. It has been reported that MES-GSC and PN-GSC are two mutually exclusive GSC subtypes with distinct dysregulated signaling pathways [46]. Unexpectedly, from the heatmap we also found two groups of genes that were distinctively expressed between resistant clones and sensitive clones; one group was downregulated and the other group was upregulated in resistant clones when compared to sensitive clones. The upregulated genes in resistant clones were mostly associated with tumor suppressor, anti-growth, anti-inflammation, anti-apoptosis, and cellular maintenance (Figure 8, Table S4), therefore functionally similar to that of the molecular signatures of resistant clones (Table 1). Conversely, the genes down-regulated in resistant clones are those with a role in tumor growth/progression, inflammation, extracellular remodeling, and immune responses (Figure 8, Table S4). Moreover, since P53 is known to be significantly mutated in PN subtype (54%) and in MES subtype but with less frequency (32%) [41], we analyzed P53 status in sensitive and resistant clones. Homozygous mutations in p53 with one gain-of-function mutation allele were detceted in all tested resistant and sensitive clones (Figure S4A). Western blot analysis further revealed that all tested clones express relatively high levels of p53 protein except D431, but higher levels of phosphorylated p53 (Ser-20) were determined in resistant clones compared to those in sensitive clones after exposure to radiation (4 Gy) (Figure S4B). This data suggests the possibility that mutated p53 may contribute to radiochemoresistance in resistant clones by an unknown pathway that sensitive clones lack. Exome sequencing analysis on a randomly selected coding regions (about 50Mb) clearly showed somatic mutations introduced by the treatment as anticipated (Figure S4C). These data thus suggest that although therapy have introduced additional genetic mutations in resistant clones, these resistant clones still maintain glioblastoma properties (also was proven by the histopathologic features of tumor in animals) and support the view that the resistant clones were generated by the clonal selection for those possessing cellular quiescence with stress resistance phenotype.

## Discussion

In this study, we seek the cellular and molecular basis of glioblastoma resistance to standard treatment. We isolated and characterized tumorigenic GSC clones that survived RT and RT+TMZ. We found that the majority of tumorigenic GSC clones derived from patient tumors are sensitive to treatment, and only a minority of clones is able to survive treatment. Importantly, we found that although cells repopulated from resistant clones are still sensitive to treatment, repeated treatment could promote the expansion of resistant clones. This may suggest the hierarchy of resistant clones. If it is true that GSC are slow-cycling and radiochemoresistant, then sensitive clones may represent a more differentiated progeny derived from resistant clones. This notion is supported by a recent study which demonstrated a hierarchy of self-renewing tumor-initiating cell type in glioblastoma and suggested that the capacities for tumor initiation need not be restricted to a uniform population of GSC [47]. Despite preserving tumorigenic potential, sensitive clones have lost stress resistance, which is likely due to undergoing proliferative differentiation and losing quiescent status as evidenced by downregulation of stem cell markers and shortening of cell cycle length. By contrast, the slower cell cycle progression in resistant clones may permit repair prior to cell division. Although CD133 may not be an obligated marker for GSC, our data support the view that it may be a marker for GSC with treatment-resistant phenotype [9]. Thus, the existence of quiescent, treatment-resistant GSC clones may explain that cancer treatment with a new drug targeting the proliferative population will always lead to the emergence of resistance. Moreover, increasing number of treatment cycles will further enrich resistant clones, by which more resistant tumor-initiating cells will accumulate, leading to an aggressive, untreatable tumor in a short period of time. Although clinically relevant dosages of TMZ have effects in treatment of glioblastoma tumors, we did not observe any obvious treatment benefits when incorporated with RT in our GSC model. This may be due to our study only focusing on the stem-like cells which all express MGMT in our model.

Treatment-resistant GSC clones appear to possess both anti-aging (stem-like cells) and superior stress resistance (resistance to radiochemotherapy) properties, and the close link between these two properties has been well-demonstrated in the naked mole-rat, the longest-living rodent [48]. To explore the protective strategies which drive the super-survivability of resistant clones, we performed a comparative analysis of genome-wide gene expression profiles in resistant clones and sensitive clones, as such will allow for discovery of molecular signatures and regulatory mechanisms operated in resistant clones that are not or less activated in sensitive clones, in an unbiased and comprehensive fashion. Surprisingly, molecular signatures of resistant clones portrayed an GR-induced “anti-stress” phenotype [26]-[33] and these results were further supported by the outcome of non-suprevised sample profiling using prediction set of 595 gene hierarchical clustering, which also unexpectedly identified the similar molecular properties in resistant clones. Both profiles portray a quiescent status of resistant clones by upregulation of genes associated with anti-growth, anti-differentiation, anti-inflammation, and tumor suppressor phenotype, which was also found in mouse tissue response to CR [49]. The siRNA knockdown experiment further suggested the importance of defense signatures of resistant clones and highlight the potential link between the metabolic transformation and radiochemoresistance. The induction of autophagy by radiation contributing to radioresistance has been demonstrated in GSC system [50]. Likewise, an increased expression of the glucose deprivation response network was also identified in breast cancer cells resistant to lapatinib, suggesting resistant cells are under nutrient stress mode [51]. Although a recent study indicated that short-term starvation can augment the efficacy of standard treatment (RT+TMZ) for glioma in the aggressive murine models of glioblastoma [52], it did not conflict with our study results since GR may enhance host's stress resistance against disease. Moreover, we also demonstrated that sensitive clones and its derived progeny (fast-growing cells) are sensitive to GR, and only resistant clones remain independent from glucose. This observation suggests that resistant clones may use a GR-like mode to maintain their cellular quiescence and force cells to switch on lipid catabolism and autophagy for energy source and subsequently activate maintenance and repair machinery for survival. Resistant clones were clonally derived from the patient tumor-derived CD133^+^ GSC cultures or from clonogenic cell-derived treatment-resistant clones by in-vitro treatment selection (treatment cycle 1 and 2), thus implying intratumoral heterogeneity and a GSC hierarchy, which could be revealed via treatment selection because of their differential treatment sensitivity. It is possible that GSC with a GR-like phenotype (resistant clones) are pre-existent quiescent cells in tumor for guarding the “tumor tissue” [53]. Although additional mutations were introduced by the treatment, they continue to produce sensitive and resistant clonogenic cells, and initiate a tumor with pathophysiologic features similar to the one initiated by sensitive clones, implying that they were unlikely solely selected through the reprogramming by the DNA-damaging agents [54].

The expression microarray data combined with functional assays support the view that radiochemoresistance of resistant clones may be associated with the activation of the SIRT1 signaling pathway, autophagy, and a global DNA repair response induced by GR-like metabolic status/adaptation, by which resistant clones reinforce cellular and genomic integrity against deadly stress. SIRT1 promotes autophagy and DNA repair activity, and maintains genomic stability [43]-[45]. It has been reported that SIRT1 contributes to telomere maintenance and increases global homologous recombination [55]. Thus, our detection of increased transcriptional levels of RAD51 in resistant clones may imply that the maintenance of telomere length and integrity is part of the defense system. Correspondingly, a 6-fold increase in transcriptional levels of early growth response-1 (EGR1) was detected in resistant clones (Table 1), and it has been shown that EGR1 promotes autophagy [56] and is required for transcription activation of SIRT1 to stimulate the expression of manganese superoxide dismutase, an antioxidant enzyme that contributes to ROS scavenging [57].

In summary, our data suggest that the emergence of radiochemoresistance may arise from the selection and expansion of quiescent GSC clones expressing GR-associated stress-resistant phenotype. Reduced glucose uptake by resistant clones may transform the metabolic adaptation of genome, leading to the constitutive activation and amplification of repair programs, which form the critical basis of radiochemoresistance. Even though radiochemotherapy could introduce additional gene mutations, molecular properties of glioblastoma origin were preserved, therefore allowing for regeneration of brain tumors in animals exhibited histopathological features similar to human glioblastoma. Direct isolation of GSC clones with GR phenotype from treated and recurred tumors would provide better evidence and a better study model since extrinsic cues from their niche may provide vital signaling for further modulating molecular properties and pathophysiology that contribute to treatment resistance. Our data support the view that the novel combination of standard treatment and a therapeutic strategy targeting the metabolic stress-induced adaptation resistance, may prevent treatment-resistant GSC clone-mediated tumor recurrence.

## Supporting Information

Figure S1 In vitro treatment of GSC cultures consisting of fractionated irradiation with or without temozolomide (TMZ). GSC received (A) radiation treatment (RT) alone (4 Gy on day 1, day 4, and day 7) or (B) concomitant TMZ (5 µM) and RT followed by adjuvant TMZ treatment (10 µM) for an additional 4 days after a 2-day break. The cell populations that formed colonies after the treatment were counted on day 14.
(TIF)

Figure S2 Treatment-resistant GSC clones contain a heterogeneous population. A. Replating cells derived from single cell-derived resistant clones showed increased clonogenic cells capable of self-renewal, proliferative differentiation and migration. B. Re-treatment of cells dissociated from a single resistant clone (E445-RT+TMZ) identified treatment-sensitive clonogenic cells (majority), treatment-resistant clonogenic cells (minority) and non-proliferative single cells (a–b). Clonogenic survivors slowly regain their capability to repopulate progeny and migrate outward from tumor spheres (c–f).
(TIF)

Figure S3 Molecular signatures and defense profiles of treatment-resistant GSC clones. A. All plots show normalized gene expression values converted into a heat map. The log2 of the fold difference is indicated by the heat map scale at the bottom. Each column is an individual sample organized into cell types and selection conditions as indicated at the top. Each row is a single probe set measurement of transcript abundance for an individual gene. Probe set signals on the expression array that were ≥2-fold increased in relative expression in treatment-resistant GSC clones compared with treatment-sensitive GSC clones. Samples were permutated 100 times by dChip and identified 53 genes (Table 1 in text) at false discovery rate (FDR) of <0.1%. B. Verification of gene expression in A. Total RNA from the indicated cells were extracted. The mRNA expression levels of indicated genes were analyzed by qtRT-PCR with specific primers. GAPDH was used as an internal control gene.
(TIF)

Figure S4 Genetic mutations in treatment-sensitive and treatment-resistant GSC clones. A. Somatic variations in TP53. B. Western blot analysis of p53. C. Count of mutations introduced by treatment with RT or RT+TMZ.
(TIF)

Table S1 Primer sequences and product sizes for semi-qtRT-PCR analysis.
(DOCX)

Table S2 Genes expressed at higher levels in RT-resistant GSC clones compared with treatment-sensitive GSC clones.
(DOCX)

Table S3 Genes expressed at higher levels in RT+TMZ-resistant GSC clones compared with treatment-sensitive GSC clones.
(DOCX)

Table S4 Distinct gene expressions in treatment-resistant GSC clones compared to treatment-sensitive GSC clones identified from predictive 595 genes.
(DOCX)
