# Supplementary figures and images for: Protective Properties of Radio-Chemoresistant Glioblastoma Stem Cell Clones Are Associated with Metabolic Adaptation to Reduced Glucose Dependence
Source: PLoS One. 2013 Nov 18;8(11):e80397. doi: 10.1371/journal.pone.0080397 (PMC3832364; doi:10.1371/journal.pone.0080397)

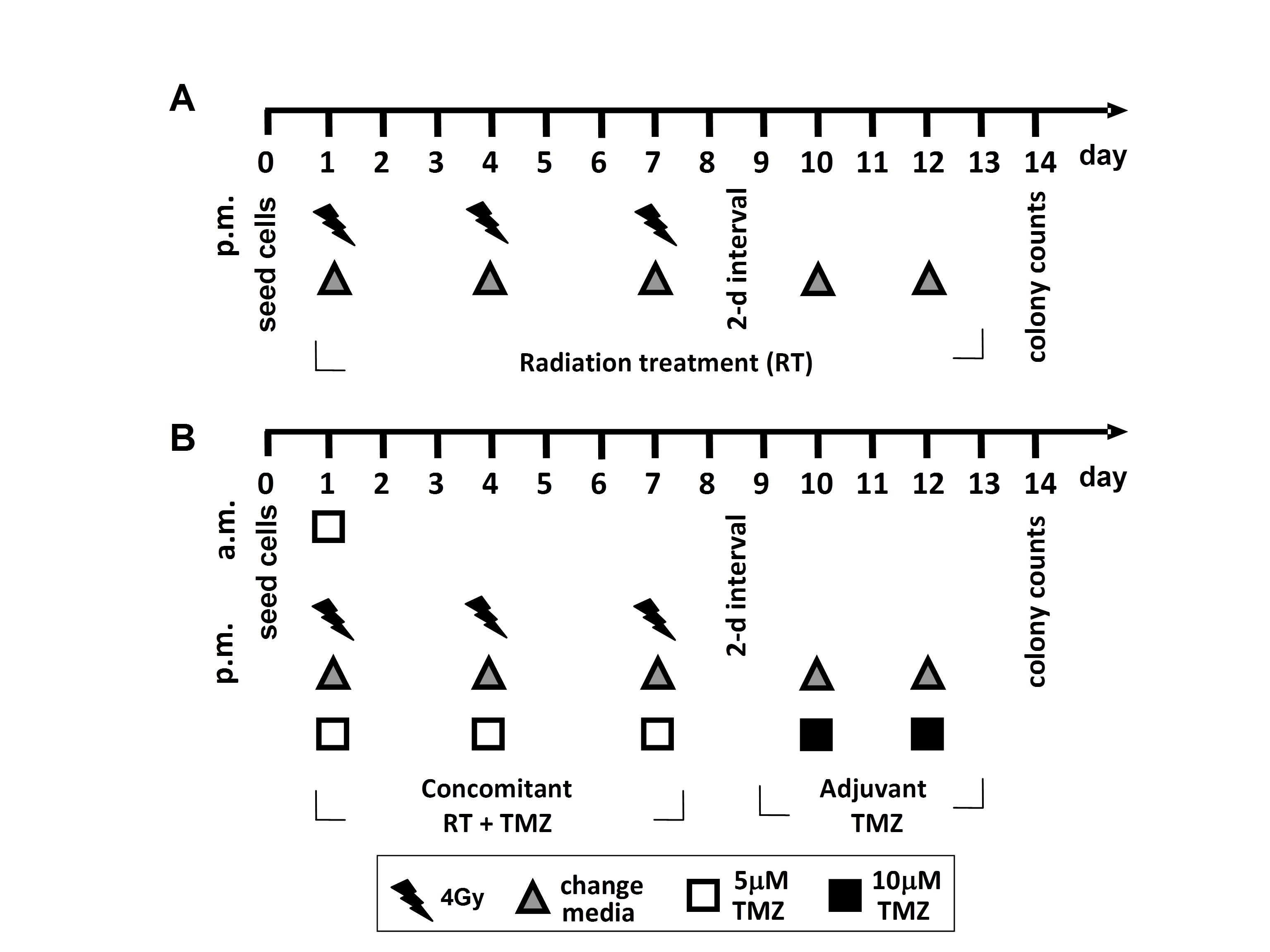

Supplement: Figure S1 — In vitro treatment of GSC cultures consisting of fractionated irradiation with or without temozolomide (TMZ). GSC received (A) radiation treatment (RT) alone (4 Gy on day 1, day 4, and day 7) or (B) concomitant TMZ (5 µM) and RT followed by adjuvant TMZ treatment (10 µM) for an additional 4 days after a 2-day break. The cell populations that formed colonies after the treatment were counted on day 14. (TIF) [file pone.0080397.s001.tif]

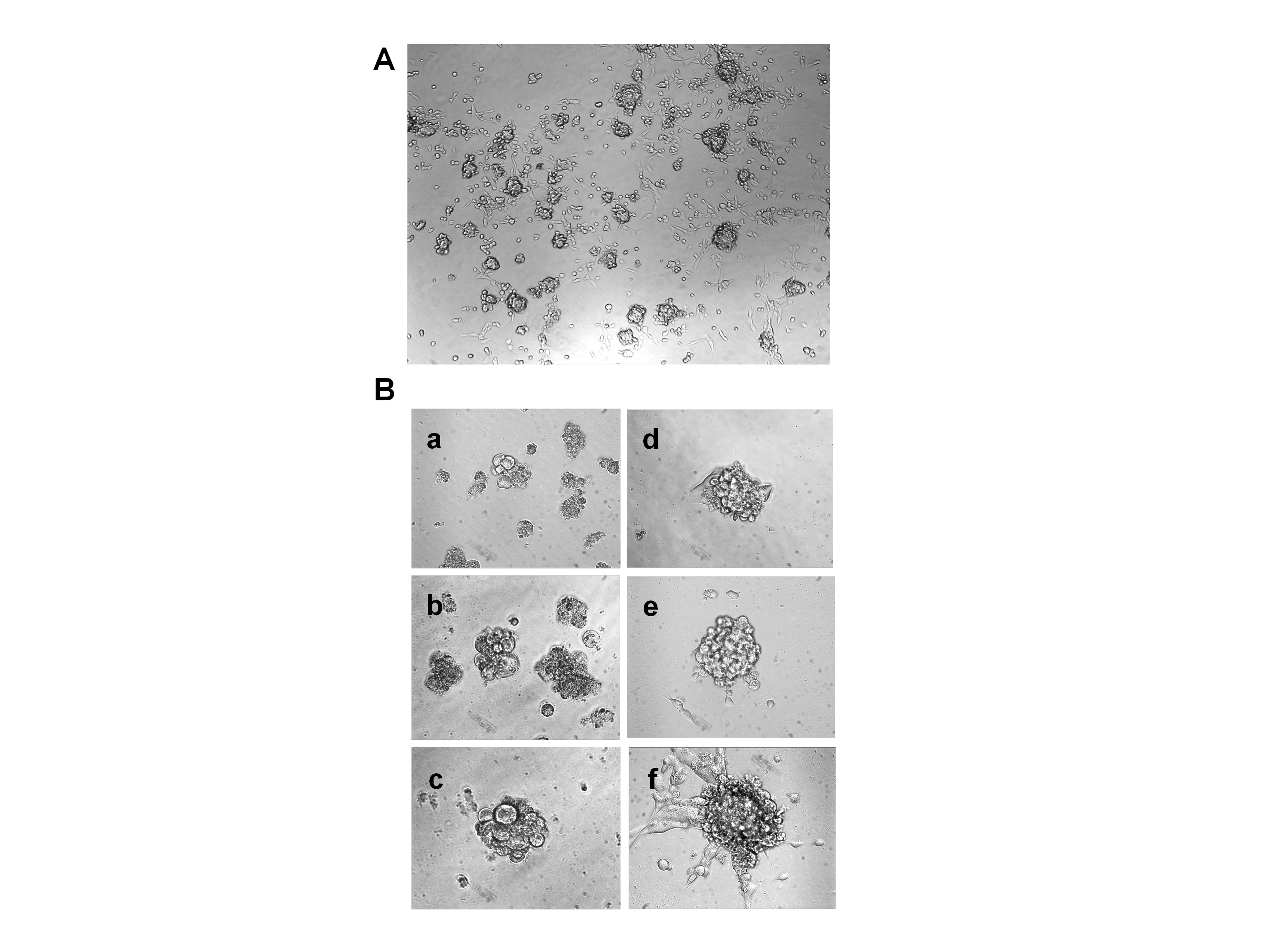

Supplement: Figure S2 — Treatment-resistant GSC clones contain a heterogeneous population. A. Replating cells derived from single cell-derived resistant clones showed increased clonogenic cells capable of self-renewal, proliferative differentiation and migration. B. Re-treatment of cells dissociated from a single resistant clone (E445-RT+TMZ) identified treatment-sensitive clonogenic cells (majority), treatment-resistant clonogenic cells (minority) and non-proliferative single cells (a–b). Clonogenic survivors slowly regain their capability to repopulate progeny and migrate outward from tumor spheres (c–f). (TIF) [file pone.0080397.s002.tif]

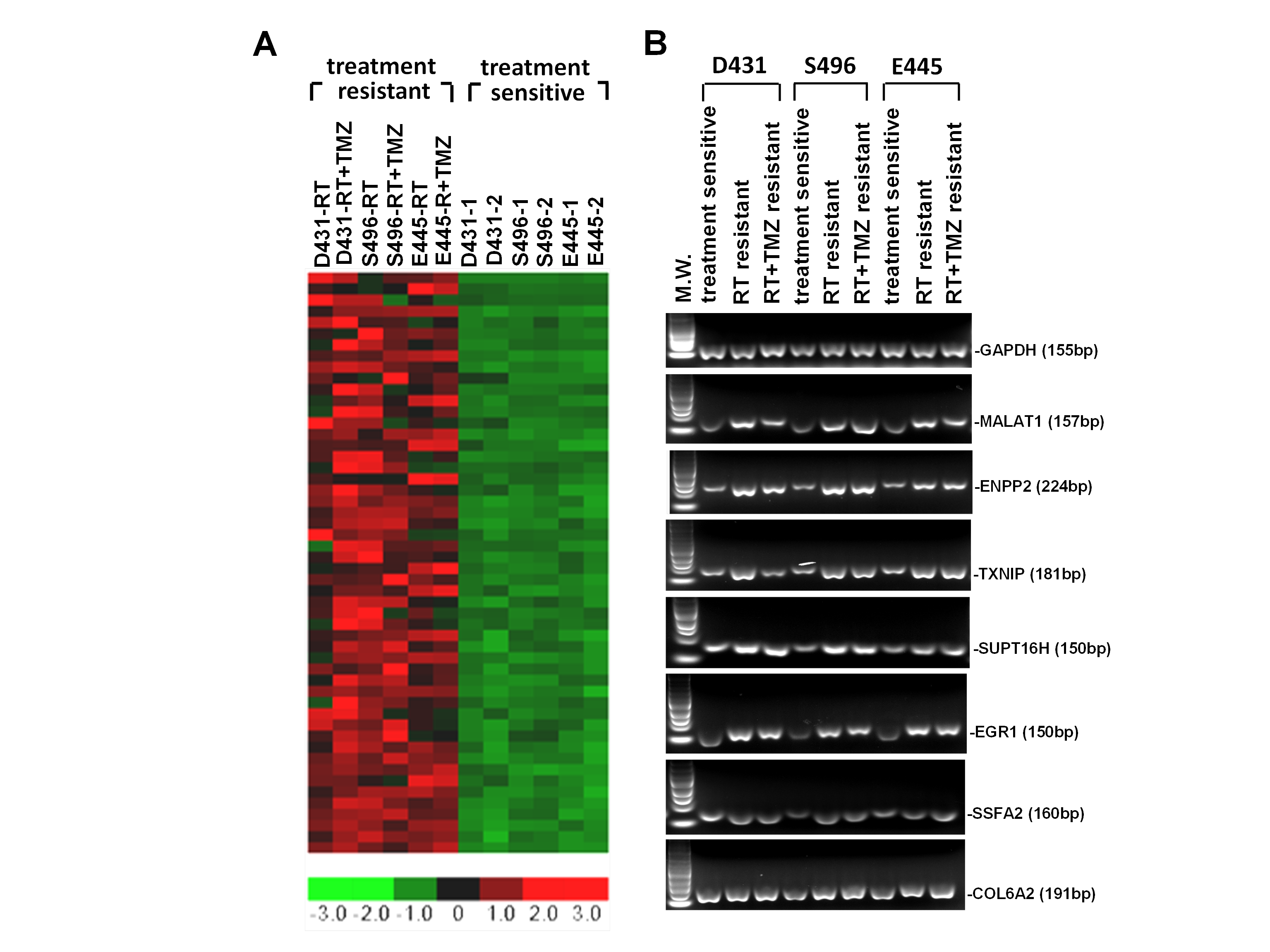

Supplement: Figure S3 — Molecular signatures and defense profiles of treatment-resistant GSC clones. A. All plots show normalized gene expression values converted into a heat map. The log2 of the fold difference is indicated by the heat map scale at the bottom. Each column is an individual sample organized into cell types and selection conditions as indicated at the top. Each row is a single probe set measurement of transcript abundance for an individual gene. Probe set signals on the expression array that were ≥2-fold increased in relative expression in treatment-resistant GSC clones compared with treatment-sensitive GSC clones. Samples were permutated 100 times by dChip and identified 53 genes (Table 1 in text) at false discovery rate (FDR) of <0.1%. B. Verification of gene expression in A. Total RNA from the indicated cells were extracted. The mRNA expression levels of indicated genes were analyzed by qtRT-PCR with specific primers. GAPDH was used as an internal control gene. (TIF) [file pone.0080397.s003.tif]

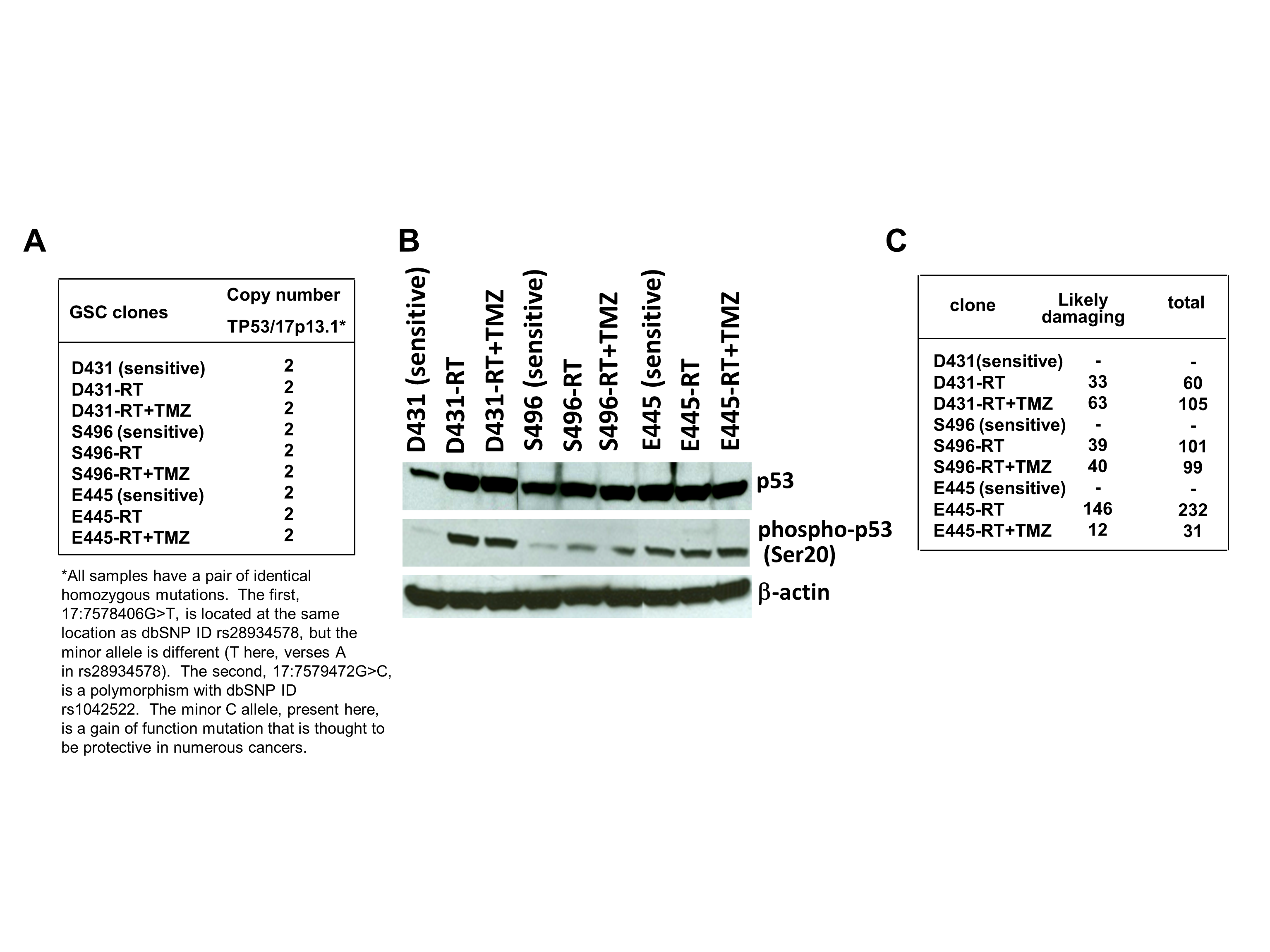

Supplement: Figure S4 — Genetic mutations in treatment-sensitive and treatment-resistant GSC clones. A. Somatic variations in TP53. B. Western blot analysis of p53. C. Count of mutations introduced by treatment with RT or RT+TMZ. (TIF) [file pone.0080397.s004.tif]
