# Supplementary material for: Protective Properties of Radio-Chemoresistant Glioblastoma Stem Cell Clones Are Associated with Metabolic Adaptation to Reduced Glucose Dependence
Source: PLoS One. 2013 Nov 18;8(11):e80397. doi: 10.1371/journal.pone.0080397 (PMC3832364; doi:10.1371/journal.pone.0080397)
Supplement: Table S1 — Primer sequences and product sizes for semi-qtRT-PCR analysis. (DOCX) [file pone.0080397.s005.docx]

Table S1. Primer sequences and product sizes for semi-qtRT-PCR analysis

|  |  |
| --- | --- |
| Gene | Primer Sequences |
|  |  |
| **ACTB** |  |
| Left Primer | ACTCAGGATTTAAAAACTGGAACG |
| Right Primer | GACTTCCTGTAACAATGCATCTCAT |
| Product Size: 152 |  |
| **GAPDH** |  |
| Left Primer | ATTTCCATTGATGACAAGCTTCC |
| Right Primer | CTGCTTTTAACTCTGGTAAAGTGGA |
| Product Size: 155 |  |
| **MAP1LC3A** |  |
| Left Primer | TGTTAGGGTTGTCCCTCTGG |
| Right Primer | GGGACAATGACCACAGATCC |
| Product Size: 235 |  |
| **MALAT1** |  |
| Left Primer | TAGGTGCTAGTTCTTGGAGTTTTG |
| Right Primer | TACCTGAAAAAGACAAGGCAGTTAT |
| Product Size: 157 |  |
| **ENPP2** |  |
| Left Primer | CCACTGTTTTTCTCTAATGCTTGAT |
| Right Primer | ACAGTTAACAGCAAATAAAGGCAAC |
| Product Size: 224 |  |
| **TXNIP** |  |
| Left Primer | CCCTGTGTTAGGAGATAGGGATATT |
| Right Primer | AATACAAAAACATGAAACCAACCAT |
| Product Size: 181 |  |
| **SUPT16H** |  |
| Left Primer | GAAAATGGTTTCCCATTTGGT |
| Right Primer | AGAGAACCAGAAGGGGAGGA |
| Product Size: 156 |  |
| **SSFA2** |  |
| Left Primer | GAAAATGGTTTCCCATTTGGT |
| Right Primer | CTGTTAACAACTCAACCACTTTTGA |
| Product Size: 160 |  |
| **COL6A2** |  |
| Left Primer | AGAGCTGTCCTTCGTGTTCCT |
| Right Primer | TGTCATAGTCCTTCTCGTGGAA |
| Product Size: 191 |  |
| **EGR1** |  |
| Left Primer | ATTCAGGAGTTGGAATGTTGTAGTT |
| Right Primer | TTCAATGTGTTTATAAGCCAAACAG |
| Product Size: 177 |  |
| **SIRT1** |  |
| Left Primer | TTTGCATGATGTTTGTGTGC |
| Right Primer | TCAAAAGTTGAAGGAAGATATCCA |
| Product Size: 177 |  |
| **PPARGC1A** |  |
| Left Primer | GCTGAAGAGGGAAAGTGAGC |
| Right Primer | AGCTCAGTGAGGCTGATGTG |
| Product Size: 150 |  |
| **FOXO1** |  |
| Left Primer | AGTCATGCCTCCTGCATTTC |
| Right Primer | AAAGAAAGACACCAAGCCATTT |
| Product Size: 183 |  |
|  |  |
| **FOXO3** |  |
| Left Primer | TCCTTCTTTCAATGCGAACA |
| Right Primer | ACATGCGTCACCATCTCTTTT |
| Product Size: 243 |  |
| **PRKAA1** |  |
| Left Primer | TGGGGCTAGATGTGGAACTC |
| Right Primer | TCTTGTCAGAACTCACAATGGAA |
| Product Size: 155 |  |
| **POLB** |  |
| Left Primer | GAAATACCGGGAACCCAAG |
| Right Primer | TTGCAAGCAAAAGCATGAAG |
| Product Size: 156 |  |
| **RAD23A** |  |
| Left Primer | CCTCTCCATCCTCCGAAAA |
| Right Primer | TCAGGACTTGCCCTAGATGG |
| Product Size: 128 |  |
| **RAD51** |  |
| Left Primer | AGCTGGGAACTGCAACTCA |
| Right Primer | TAGGCAACAGCCTCCACAG |
| Product Size: 230 |  |
| **Ku70/XRCC6** |  |
| Left Primer | AGAGGAGCTGAAGACCCACA |
| Right Primer | CCTCTGGTCAGTCCTGGAAG |
| Product Size: 155 |  |
| **BECN1** |  |
| Left Primer | CTCCCGAGGTGAAGAGCAT |
| Right Primer | GTTCCTGGATGGTGACACG |
| Product Size: 156 |  |
| **PROM1/CD133** |  |
| Left Primer | AAACATTAATTGGAGTGCAGCTAAC |
| Right Primer | TCTTCATTGCTGATCACTTTTGATA |
| Product Size: 226 |  |
| **SOX2** |  |
| Left Primer | CCAACTTTCCATTTTGTTCAGATAA |
| Right Primer | CAGAGCCGAATCTTTTAAAATACAA |
| Product Size: 155 |  |
| **MSI1** |  |
| Left Primer | TAATTGTCTGTGTGAGGTGCTTAAC |
| Right Primer | GACTCAGCTCAAACGTGTAGAAAAT |
| Product Size: 180 |  |
| **MELK** |  |
| Left Primer | GTTCATTGGAACTACCAACTTGTTT |
| Right Primer | TGGAAGTGAATCTAAGAAAGTCAACA |
| Product Size: 185 |  |
| **SOX4** |  |
| Left Primer | GCAATATGCCGTGTAGAATATTTGT |
| Right Primer | CAGCTGATTTTATTTCCTTCTCAAA |
| Product Size: 172 |  |
| **NES** |  |
| Left Primer | CTAAGTCAGCTGAATCCCGATAGTA |
| Right Primer | GTGCTACTGCAGTGAGATGGTG |
| Product Size: 178 |  |
