# Supplementary material for: Protective Properties of Radio-Chemoresistant Glioblastoma Stem Cell Clones Are Associated with Metabolic Adaptation to Reduced Glucose Dependence
Source: PLoS One. 2013 Nov 18;8(11):e80397. doi: 10.1371/journal.pone.0080397 (PMC3832364; doi:10.1371/journal.pone.0080397)
Supplement: Table S2 — Genes expressed at higher levels in RT-resistant GSC clones compared with treatment-sensitive GSC clones. (DOCX) [file pone.0080397.s006.docx]

Table S2. Genes expressed at higher levels in RT-resistant GSC clones compared with treatment-sensitive GSC clones

| Gene Name and Gene Symbol | Fold Change | P-Value |
| --- | --- | --- |
| MALAT1: metastasis associated lung adenocarcinoma transcript 1 | 15.15 | 0.02861 |
| SUPT16H: suppressor of Ty 16 homolog (S. cerevisiae) | 6.19 | 0.02737 |
| EPRS: glutamyl-prolyl-tRNA synthetase | 5.71 | 0.03593 |
| IL6ST: interleukin 6 signal transducer (gp130, oncostatin M receptor) | 4.99 | 0.01741 |
| C5orf24: chromosome 5 open reading frame 24 | 4.40 | 0.03905 |
| MFAP4: microfibrillar-associated protein 4 | 4.08 | 0.00594 |
| RPS11: Ribosomal protein S11 | 3.76 | 0.03092 |
| ZC3H11A: zinc finger CCCH-type containing 11A | 3.49 | 0.00641 |
| TTC3: tetratricopeptide repeat domain 3 | 3.40 | 0.00814 |
| SLC38A1: solute carrier family 38, member 1 | 3.36 | 0.03754 |
| TPM4: tropomyosin 4 | 3.25 | 0.02198 |
| RPL38: ribosomal protein L38 | 3.10 | 0.01596 |
| CPNE3: copine III | 3.10 | 0.00636 |
| MATR3: matrin 3 | 3.01 | 0.02550 |
| NBPF family: neuroblastoma breakpoint family, members | 3.00 | 0.04679 |
| PRKCI: protein kinase C, iota | 2.88 | 0.03931 |
| HSP90B1: heat shock protein 90kDa beta (Grp94), member 1 | 2.85 | 0.01720 |
| BAT2D1: BAT2 domain containing 1 | 2.78 | 0.02537 |
| PRPF6: PRP6 pre-mRNA processing factor 6 homolog (S. cerevisiae) | 2.60 | 0.01646 |
| HTRA1: HtrA serine peptidase 1 | 2.54 | 0.02952 |
| PPP2R1A: protein phosphatase 2 (formerly 2A), regulatory subunit A, alpha isoform | 2.36 | 0.00431 |

NOTE: Probe set signals on the expression array that were ≥2-fold increased in relative expression in treatment-resistant GSC clones against radiation treatment (RT) (n= 3 clones from 3 patients) compared with treatment-sensitive GSC clones (n=6 clones from 3 patients) were selected. Samples were permutated 100 times by dChip and identified 21 genes at false discovery rate (FDR) of 3.8%.
