# Supplementary material for: Protective Properties of Radio-Chemoresistant Glioblastoma Stem Cell Clones Are Associated with Metabolic Adaptation to Reduced Glucose Dependence
Source: PLoS One. 2013 Nov 18;8(11):e80397. doi: 10.1371/journal.pone.0080397 (PMC3832364; doi:10.1371/journal.pone.0080397)
Supplement: Table S3 — Genes expressed at higher levels in RT+TMZ-resistant GSC clones compared with treatment-sensitive GSC clones. (DOCX) [file pone.0080397.s007.docx]

Table S3. Genes expressed at higher levels in RT+TMZ-resistant GSC clones compared with treatment-sensitive GSC clones

| Gene Name and Gene Symbol | Fold Change | P-Value |
| --- | --- | --- |
| MALAT1: metastasis associated lung adenocarcinoma transcript 1 | 9.94 | 0.049809 |
| SUPT16H: suppressor of Ty 16 homolog (S. cerevisiae) | 7.22 | 0.000269 |
| IL6ST: interleukin 6 signal transducer (gp130, oncostatin M receptor) | 5.63 | 0.014861 |
| COL6A2: collagen, type VI, alpha 2 | 5.12 | 0.031525 |
| EGR1: early growth response 1 | 5.06 | 0.037718 |
| PLEKHA2: pleckstrin homology domain containing, family A | 4.43 | 0.030861 |
| C5orf24: chromosome 5 open reading frame 24 | 4.34 | 0.040999 |
| SRPR: signal recognition particle receptor ('docking protein') | 4.10 | 0.004159 |
| TPM4: tropomyosin 4 | 3.94 | 0.009350 |
| RASSF8: Ras association (RalGDS/AF-6) domain family 8 | 3.65 | 0.039452 |
| MATR3: matrin 3 | 3.57 | 0.001625 |
| RPS11: Ribosomal protein S11 | 3.55 | 0.023412 |
| ZFR: zinc finger RNA binding protein | 3.54 | 0.034652 |
| ZC3H11A: zinc finger CCCH-type containing 11A | 3.51 | 0.045788 |
| NBPF family: neuroblastoma breakpoint family, members | 3.46 | 0.045692 |
| PDLIM5: PDZ and LIM domain 5 | 3.46 | 0.018921 |
| NR2F1: nuclear receptor subfamily 2, group F, member 1 | 3.45 | 0.042579 |
| RPL38: ribosomal protein L38 | 3.12 | 0.010745 |
| UBA6: ubiquitin-like modifier activating enzyme 6 | 3.10 | 0.002049 |
| RAB12: RAB12, member RAS oncogene family | 3.04 | 0.042511 |
| NR2F1: Nuclear receptor subfamily 2, group F, member 1 | 2.76 | 0.043794 |
| SF3B1: splicing factor 3b, subunit 1, 155kDa | 2.75 | 0.010585 |
| PHF3: PHD finger protein 3 | 2.67 | 0.004128 |
| OSBPL8: oxysterol binding protein-like 8 | 2.60 | 0.009795 |
| PRPF6: PRP6 pre-mRNA processing factor 6 homolog (S. cerevisiae) | 2.51 | 0.018756 |
| AGGF1: angiogenic factor with G patch and FHA domains 1 | 2.50 | 0.004046 |
| MOBKL1B: MOB1, Mps One Binder kinase activator-like 1B (yeast) | 2.47 | 0.019228 |

NOTE: Probe set signals on the expression array that were ≥2-fold increased in relative expression in treatment-resistant GSC clones against radiation treatment (RT) plus temozolomide (TMZ) (n= 3 clones from 3 patients) compared with treatment-sensitive GSC clones (n=6 clones from 3 patients) were selected. Samples were permutated 100 times by dChip and identified 27 genes at false discovery rate (FDR) of 4.8%.
