# Supplementary material for: Protective Properties of Radio-Chemoresistant Glioblastoma Stem Cell Clones Are Associated with Metabolic Adaptation to Reduced Glucose Dependence
Source: PLoS One. 2013 Nov 18;8(11):e80397. doi: 10.1371/journal.pone.0080397 (PMC3832364; doi:10.1371/journal.pone.0080397)
Supplement: Table S4 — Distinct gene expressions in treatment-resistant GSC clones compared to treatment-sensitive GSC clones identified from predictive 595 genes. (DOCX) [file pone.0080397.s008.docx]

Table S4. Distinct gene expressions in treatment-resistant GSC clones compared to treatment-sensitive GSC clones identified from predictive 595 genes

| Gene Symbol | Functional Involvement |
| --- | --- |
|  |  |
| Upregulated genes |  |
| FSTL1 | promotes endothelial cell function and stimulates revascularization |
| EMP3 | tumor suppressor |
| HTATIP2 | tumor suppressor |
| ZNF488 | oligodendrocyte development; transcription regulation |
| REPS2 | negatively affect receptor internalization and inhibit growth factor signaling; anti-growth |
| NRG3 | a survival factor for oligodendrocytes |
| NCF2 | superoxide production |
| MARCKS | cell motility; phagocytosis; membrane trafficking; mitogenesis |
| IL1R2 | anti-inflammation |
| MAP3K8 | tumor suppressor; antiapoptosis; stress-activated protein kinase |
| BRUNOL4 | tumor suppressor gene |
| MAP4 | promotes microtubule assembly; stabilizes mitochondria; cell viability |
| H19 | an imprinted gene; regulate IGF2; tumor suppressor; mesenchymal-to-epithelial transition |
| FCGR2B | blocks inflammatory immune response |
| ZEB1 | transcriptional repressor; inhibits IL-2 gene expression; induces an epithelial-mesenchymal transition |
| SLC7A1 | transports cationic amino acids; drug resistance |
| TIMP1 | inhibitors of the matrix metalloproteinases; antiapoptosis |
| FNDC3B | adipogenesis; driver for clonogenicity and tumorigenicity |
| RNF135 | involved in innate immune defense against viruses, promotes interferon beta production; close link to NF1 |
| LOC727942 | unknown |
| C1orf162 | unknown |
| NID2 | maintaining the structure of the basement membrane |
| KCNIP3 | calcium-regulated transcriptional repressor; cell cycle arrest |
| ZDHHC22 | putative palmitoyl-transferases; controls surface expression of calcium-activated potassium channels |
|  |  |
| Downregulated genes |  |
| NAPSB | stimulates chemotaxis; express in immune related cells |
| FXYD1 | phosphorylated in response to insulin and adrenergic stimulation |
| SYT13 | membrane traffickers |
| RASL10A | transports vesicle docking to the plasma membrane |
| IGF2 | an imprinted gene; development and growth; 5' region overlaps the insulin gene; regulated by H19 |
| PDE8B | hydrolyzes the second messenger cAMP |
| CRTAC1 | cell-cell or cell-matrix interactions; chondrogenic differentiation |
| F13A1 | thrombosis and wound healing; stabilizing the fibrin clot |
| SCN2B | functional modulation of the heterotrimeric complex of the sodium channel |
| MMP9 | local proteolysis of the extracellular matrix; tumor-associated tissue remodeling |
| STAB1 | clearance of apoptotic cells |
| BCAN | promotes the growth and cell motility of brain tumor; invasiveness of low-grade astrocytoma |
| FAM123A | decreases actomyosin contractility; a negative regulator of Wnt signaling |
| STX1B | excitatory pathway of synaptic transmission |
| HRNBP3 | RNA-binding protein that regulates alternative splicing events |
| IL1R1 | induces immune and inflammatory responses |
| RASGRF2 | required for IL-1 induction of the Ras→ERK pathway and MMP-3 expression |
| SVOP | neuron formation, maturation, or neuronal function |
| S100A4 | tumor progression; angiogenesis; metastasis |
| RCAN2 | upregulated by thyroid hormone; endothelial cell function and angiogenesis |
| CKS2 | cell cycle progression; tumorigenesis |
| ZNF367 | transcriptional activation of erythroid genes |
| BCL11A | leukemogenesis and hematopoiesis |
| SPON1 | vascular smooth muscle growth-promoting factor |
| MS4A6A | displays unique expression patterns among hematopoietic cells and non-lymphoid tissues |
| SERPING1 | regulates complement activation |
| SYT4 | vesicular trafficking and exocytosis |
| EDNRB | activation of vascular smooth muscle cells and endothelial cells |
| FCGR3A | mediates antibody-dependent responses |

NOTE: Selection of upregulated and downregulated gene lists was based on the results of heat map in Figure 8.
